# Decision algorithm for picture fuzzy sets and Aczel Alsina aggregation operators based on unknown degree of wights

**DOI:** 10.1016/j.heliyon.2024.e27548

**Published:** 2024-03-10

**Authors:** Abrar Hussain, Yu Liu, Kifayat Ullah, Muhammad Rashid, Tapan Senapati, Sarbast Moslem

**Affiliations:** a Department of Mathematics, Riphah International University (Lahore Campus), 54000, Lahore, Pakistan; b College of Economics and Management, Hebei Agricultural University, Baoding, 071001, China; c School of Mathematics and Statistics, Southwest University, Beibei, 400715, Chongqing, China; d School of Architecture Planning and Environmental Policy, University College of Dublin, D04 V1W8, Belfield, Dublin, Ireland

**Keywords:** Aczel Alsina aggregation tools, Power average operator, Picture fuzzy numbers, and multi-attribute group decision-making technique

## Abstract

Aggregation operators (AOs) are well-known and efficient mathematical tools that are utilized to overcome the impact of imprecise and vague information during the aggregation process. The theoretical concepts of Aczel Alsina aggregation expressions are an extension of triangular norms and become a hot research topic in the environment of the fuzzy framework. The power operators provide a smooth approximation and are used to mitigate the influence of redundant or insufficient information on the attributes or criteria. Some robust aggregation approaches are developed by combining two different theories, like power operators and Aczel Alsina aggregation tools. This article aims to explore the theory of picture fuzzy sets (PFSs), an extended version of fuzzy sets, and intuitionistic fuzzy sets. Some robust operations of Aczel Alsina aggregation tools are also present in light of the picture fuzzy environment. We established a class of new methodologies in the light of picture fuzzy information, including picture fuzzy Aczel Alsina power weighted average (PFAAPWA) and picture fuzzy Aczel Alsina power ordered weighted average (PFAAPOWA) operators. We also developed an appropriate approach like picture fuzzy Aczel Alsina power weighted geometric (PFAAPWG) and picture fuzzy Aczel Alsina power ordered weighted geometric (PFAAPOWG) operators. Notable properties and characteristics of proposed methodologies are also demonstrated. Our invented approaches not only aggregate complicated information but can clearly define interrelationships among several arguments. Moreover, we establish an algorithm for the multi-attribute group decision-making (MAGDM) problem to handle the impact of redundant and vague information on human opinions. Finally, we study an experimental case study to evaluate an appropriate optimal option from available options. To reveal consistency and effectiveness of developed approaches, influence study by changing various parametric values and comparative study by comparing results of existing approaches.

## 1 Introduction

The multi-attribute group decision-making (MAGDM) problem is a modified form of an advanced decision-making technique and a dominant strategy for demonstrating the valuable predilection from the collection of preferences, including those of different citizen groups and stakeholders. Numerous experts evaluate real-life problems in our daily lives and try to find a reliable, optimal option under certain criteria or characteristics. Generally, the expert presents conventional knowledge without assessing its level of ambiguity and uncertainty [[1], [2], [3], [4], [5], [6], [7], [8], [9]]. Even though one of the most useful and deserving hypotheses for expressing the positive value (PV) of information whose range lies in close interval [0,1]. The theory of fuzzy sets (FSs) developed by Zadeh [10] is one of the most genuine and well-meaning concepts for treating unpredictable and vague information. The FSs were extensively used in a variety of contexts, including [11,12]. Many people have asked where the information is false in this case, raising that concern. To address the aforementioned issue, Atanassov [13] developed the theory of intuitionistic FSs (IFSs), which effectively characterizes the PV and negative value (NV) of the information whose range is in a unit interval and has the distinguishing property 0≤δ+ζ≤1. Although the theory of IFSs is a generalized version of FSs, it is not without limitations. For example, during an election, experts have encountered four different sorts of problems, including yes, abstinence, no, and neutral or refusal, where the theory of IFSs has been disregarded to address some issues [14]. To address the aforementioned issues, Cuong [15] developed the theory of picture FSs (PFSs), which is a commendable concept that describes the PV, abstinence value (AV), and negative value (NV) of information whose range is in a unit interval with a conspicuous feature 0≤δ+ç+ζ≤1, where the dynamic distance measurements were produced in Ref. [16].

In decision-making, AOs play an effective role in the aggregation process. Many scholars developed different AOs to handle dubious imprecision information. Arora and Garg [17] explored the properties of prioritized aggregation models based on linguistic FSs and developed new mathematical approaches to solve advanced decision-making approaches. Xu and Yager [18,19] produced some appropriate aggregation models by exploring the speculation of the arithmetic and geometric operators. Kaur and Garg [20] anticipated certain approaches of cubic IFSs, which are the robust concepts of IFSs, and utilized them to produce some new methodologies. Akram et al. [21] illustrated certain characteristics of Dombi aggregation tools and developed new approaches to evaluate a MADM problem. Ullah [22] characterized some new approaches to PFSs by exploring the theory of symmetric mean aggregation operators. Hussain et al. [23] determined the correlation among input arguments based on complex IFSs and established a MADM problem to assess a suitable tourism industry. Riaz and Farid [24] expressed the superiority of the PFSs and investigated some new approaches to complete the selection process under the third party of the linguistic provider. Ali et al. [25] explored the theory of the decision-making process under the extended fuzzy framework. Khan et al. [26] stated some new approaches to IFSs by utilizing the speculative theory of the Schweizer-Sklar tools and power aggregation models. Rahim et al. [27] imitated some trigonometric functions to develop some new methodologies based on cubic PyF information. Hussain et al. [28] investigated a reliable energy resource under consideration of an intuitionistic fuzzy framework. Ai et al. [29] generalized the continuous Archimedean triangular norms theory to handle uncertain imprecision information in decision-making. Riaz et al. [30] anticipated some appropriate aggregation mathematical tools based on Einstein-prioritized AOs under the system of q-rung orthopair FSs.

Menger produced the hypothetical concepts of triangular norms in the circumstances of statistical metric space [31]. The theory of triangular norms has been found more broadly and presented certain operations in different fuzzy environments such as Algebraic triangular norms [32], product triangular norms [33], probabilistic triangular norms [34], Dombi triangular norms [35], Einstein triangular norm [36], Hamacher triangular norms [37], Lukasiewicz triangular norms [38], Frank triangular norms [39], Nilpotent triangular norms [40] and so far. In a few decades, numerous scientists have collected extensive information by exploring the primary characteristics of triangular norms and produced some innovative operational laws of triangular norms. Ali and Mahmood [41] developed some new strategies for complex q-ROFSs using basic Dombi aggregation tools operations. Seikh and Mandal [42] utilized Dombi operational laws to mitigate the impact of insufficient information based on some specific attributes under the system of an intuitionistic fuzzy framework. Liang et al. [43] expressed a well-known algorithm to evaluate a MADM problem by utilizing the Bonferroni mean tools based on PyF information. Ali et al. [44] established a MADM problem to choose an emergency selection process based on complex IF environments. Mandal and Seikh [45] presented an algorithm of the decision analysis for interval-valued spherical fuzzy information. Seikh and Mandal [46] also examined the impact of bio-medical waste materials under the interval-valued Fermatean fuzzy information system.

The extension of triangular norms in the form of Aczel Alsina aggregation tools was launched by Aczel and Alsina [47] in 1982. Recently, Aczel Alsina aggregation tools attained a lot of attention from numerous research scientists and played an effective role in the decision-making process. Farahbod and Eftekhari [48] utilized two concepts of confidence and support measures to evaluate reliable aggregation tools. After investigation, they concluded that Aczel Alsina aggregation tools are superior to others. Keeping in mind the significance of Aczel Alsina aggregation tools and their properties, Senapati et al. [49] designed certain aggregation tools to evaluate a MADM technique by utilizing the theoretic concepts of Aczel Alsina tools based on IFSs. Hussain et al. [50] derived some new approaches to Pythagorean FSs (PyFSs) and also explored the characteristics of Aczel Alsina aggregation tools. Ali et al. [51] generalized the theory of Einstein aggregation expressions and presented some appropriate mathematical approaches. Hussain et al. [52] also produced some new approaches of interval-valued PyFSs (IVPyFSs) to enhance the evaluation capability of the decision-making process based on Aczel Alsina aggregation models. Hussain et al. [53] combined two different theories, Hamy mean aggregation models and Aczel Alsina aggregation tools under a system of intuitionistic fuzzy information. Naeem et al. [54] presented some major characteristics of Aczel Alsina aggregation tools to illustrate a list of new approaches based on PFSs. Moreover, adopting fuzzy logic in evaluating complex problems has been conducted in several research areas [[55], [56], [57]].

The PFS is an efficient and powerful aggregation model, which is the extended form of FSs and IFSs with three components of human opinions: positive value (PV), abstain value (AV), and negative value (NV). The power operators are used to mitigate the impact of redundant information of human opinions and also express relationships among different input arguments. Aczel Alsina aggregation tools are more flexible aggregation models and can provide authentic estimated information during the decision-making process. Considering the significance of Aczel Alsina aggregation tools and Power operators, we developed some robust methodologies in the light of PF information to investigate an appropriate optimal option by combining two concepts, power operators and Aczel Alsina aggregation tools. The key components of the proposed research work are demonstrated as follows:

a) To expose notions of PFSs and their related fundamental operations, which are used for further development of presented research work.
b) To deduce some realistic operations of Aczel Alsina aggregation tools in the light of PF information.
c) The concepts of power operators are used to reduce the influence of redundant information and also define relationships among different input arguments.
d) We derived a class of new approaches by combining two different theories under considering PF environments like PFAAPWA, PFAAPOWA, PFAAPWG, and PFAAPOWG operators.
e) e) To show the reliability and feasibility of the proposed methodologies, we also presented some notable properties and characteristics.
f) We also illustrated an algorithm of the MAGDM problem to resolve an application of real-life problems based on PF information. To reveal the intensity and effectiveness of invented approaches, we gave an experimental case study to choose a desirable optimal option by utilizing proposed methodologies.
g) To see the supremacy and superiority of our proposed approaches, compare the findings of current techniques with existing methodologies.

The outline of this manuscript is organized as follows: Section 2 covers basic concepts of triangular norms, Aczel Alsina aggregation tools, PFSs, and their related basic operations. Some reliable operations of Aczel Alsina triangular norms are also illustrated in section 3. Section 4 demonstrated a class of new approaches by combining two different theories, such as Aczel Alsina aggregation tools and power operators, such as the PFAAPWA operator, with realistic characteristics and some special cases. Section 5 also derived a list of new mathematical approaches like PFAAPWG and PFAAPOWG operators. Section 6 ratifies the intensity of our derived approaches by exploring the robust algorithm of the MAGDM problem under the system of PF information. To show the superiority of the developed approach, we also gave an experimental case study to resolve real-life dilemmas. To explore the performance and flexibility of derived approaches, a comparison technique is also presented in section 7. Section 8 defines some remarkable remarks related to our research work.

## 2 Preliminaries

We elaborated on some notions of triangular norms, Aczel Alsina aggregation tools, PFSs, and their related appropriate operations for improving this manuscript.

Definition 1 [31] A mapping Β:[0,1]×[0,1]⟶[0,1] is said to be t-norm, if it fulfils the following condition:

i. Β(ǟ,ğ)=(ğǟ).
ii. Β(ǟ,ğ)≤Β(ǟ,ç) If ǟ≤ç.
iii. Β(ǟ,Β(ǟ,ç))=Β(Β(ǟ,ğ),ç).
iv. Β(ǟ,1)=ǟ.

For ǟ,ğ,ç∈[0,1].

Definition 2 [31] A mapping Β:[0,1]×[0,1]⟶[0,1] is said to be t-conorm, if it fulfils the following condition:

i. A(ǟ,ğ)=(ğǟ).
ii. A(ǟ,ğ)≤A(ǟ,ç) If ǟ≤ç.
iii. A(ǟ,A(ǟ,ç))=A(A(ǟ,ğ),ç).
iv. A(ǟ,0)=ǟ.

For ǟ,ğ,ç∈[0,1].

Definition 3 [47] A mapping ${\left({Β}_{\lambda }^{\tau }\right)}_{\tau \in \left[0,\infty \right]}$ is known as Aczel - Alsina t-norm, if:

$${Β}_{\lambda }^{\tau }\left(ǟ,ğ\right)=\left\{ \begin{array}{c} {Β}_{D}\left(ǟ,ğ\right)\;If\;\tau =0\;\\ \min\left(ǟ,ğ\right)\;If\;\tau =\infty \\ {\;e}^{-{\left({\left(-\log\left(ǟ\right)\right)}^{\tau }+{\left(-\log\left(ğ\right)\right)}^{\tau }\right)}^{1/\tau }}\;otherwise \end{array}\right.$$

Definition 4 [47] A mapping ${\left(A_{\lambda }^{\tau }\right)}_{\tau \in \left[0,\infty \right]}$ is known as Aczel - Alsina t-conorm, if:

$$A_{\lambda }^{\tau }\left(ǟ,ğ\right)=\left\{ \begin{array}{cc} \begin{array}{c} A_{D}\left(ǟ,ğ\right)\\ \max\left(ǟ,ğ\right)\\ {\;1-\;e}^{-{\left({\left(-\log\left(1-ǟ\right)\right)}^{\tau }+{\left(-\log\left(1-ğ\right)\right)}^{\tau }\right)}^{1/\tau }} \end{array} & \begin{array}{c} If\;\tau =0\\ If\;\tau =\infty \\ otherwise \end{array} \end{array}\right.$$

Definition 5 [15] Consider Y to be a non-empty set and a PES K on Y is defined as:

$$K=\left\{y,\left\langle \delta_{K}\left(y\right),{ç}_{K}\left(y\right),\zeta_{K}\left(y\right)\right\rangle |y\in Y\right\}$$

Where $\delta_{K}\left(y\right)\in \left[0,1\right]$ denotes the positive value (PV) of $K,{ç}_{K}\;\left(y\right)\in \left[0,1\right]$ the abstinence value (AV) of K and ζ(y)∈[0,1] the negative value (NV) of K. A PFS must satisfy the following conditions:

$$0\leq \delta_{K}\left(y\right)+{ç}_{K}\left(y\right)+\zeta_{K}\left(y\right)\leq 1$$

A refusal value of K in Y is denoted by $\tau_{K}\left(y\right)=1-\left(\delta_{K}\left(y\right)+{ç}_{K}\left(y\right)+\zeta_{K}\left(y\right)\right)$. A picture fuzzy value (PFV) is indicated by the $K=\left(\delta_{K},{ç}_{K},\zeta_{K}\right)$.

Definition 6 [58] Suppose $K_{ȴ}=\left(\delta_{K_{ȴ}},{ç}_{K_{ȴ}},\zeta_{K_{ȴ}}\right),\left(ȴ=1,2\right)$ be any two PFVs. Then, we have:

i. $K_{1}\subseteq K_{2}\;Iff\;\delta_{K_{1}}\leq \delta_{K_{2}},{ç}_{K_{1}}\leq {ç}_{K_{2}}$ and $\zeta_{K_{1}}\geq \zeta_{K_{2}}$ for all y∈Y.
ii. $K_{1}=K_{2}\;If$$K_{1}\subseteq K_{2}$ and $K_{2}\subseteq K_{1}$.
iii. $K_{1}\cap K_{2}=\left\{\min\left(\delta_{K_{1}},\delta_{K_{2}}\right),mix\left({ç}_{K_{1}},{ç}_{K_{2}}\right),\max\left(\zeta_{K_{1}},\zeta_{K_{2}}\right)\right\}$.
iv. $K_{1}\cup K_{2}=\left\{\max\left(\delta_{K_{1}},\delta_{K_{2}}\right),\min\left({ç}_{K_{1}},{ç}_{K_{2}}\right),\min\left(\zeta_{K_{1}},\zeta_{K_{2}}\right)\right\}$.
v. ${K_{1}}^{c}=\left\{\zeta_{K_{1}},{ç}_{K_{1}},\delta_{K_{1}}\;\right\}$.

Definition 7 [59] Suppose $K_{ȴ}=\left(\delta_{K_{ȴ}},{ç}_{K_{ȴ}},\zeta_{K_{ȴ}}\right),\left(ȴ=1,2\right)$ be any two PFVs. Then, some operations are defined as follows:

i. $K_{1}⨁K_{2}=\left\{\delta_{K_{1}}+\delta_{K_{2}}-\delta_{K_{1}}\delta_{K_{2}},{ç}_{K_{1}}{ç}_{K_{2}},\zeta_{K_{1}}\zeta_{K_{2}}\right\}$.
ii. $K_{1}⨂K_{2}=\left\{\begin{array}{c} \delta_{K_{1}}\delta_{K_{2}},{ç}_{K_{1}}+{ç}_{K_{2}}-{ç}_{K_{1}}{ç}_{K_{2}}\text{,}\\ \zeta_{K_{1}}+\zeta_{K_{2}}-\;\zeta_{K_{1}}\zeta_{K_{2}} \end{array}\right\}$.
iii. $\tau {.K}_{1}=\left\{1-{\left(1-\delta_{K_{1}}\right)}^{\tau },{\left({ç}_{K_{1}}\right)}^{\tau },{\left(\;\zeta_{K_{1}}\right)}^{\tau }\right\},\tau >0$.
iv. ${K_{1}}^{\tau }=\left\{\begin{array}{c} {\left(\delta_{K_{1}}\right)}^{\tau },1-{\left(1-{ç}_{K_{1}}\right)}^{\tau }\text{,}\\ \;1-{\left(1-\zeta_{K_{1}}\right)}^{\tau } \end{array}\right\},\tau >0$.

Definition 8 [59] Suppose $K=\left(\delta_{K_{ȴ}},{ç}_{K_{ȴ}},\zeta_{K_{ȴ}}\right)$ be a PFV. The score function S(K) and accuracy function U(K) of PFV is expressed in the following Equation (1) and Equation (2) respectively:

(1) $$S\left(K\right)=\frac{1}{2}\left(1+\left(\delta_{K_{1}}-\zeta_{K_{1}}\right)\right)$$

and

(2) $$U\left(K\right)=\left(\delta_{K_{1}}+{ç}_{K_{1}}+\zeta_{K_{1}}\right)$$

Where S(K)∈[0,1] and U(K)∈[0,1].

Definition 9 Suppose $K_{ȴ}=\left(\delta_{K_{ȴ}},{ç}_{K_{ȴ}},\zeta_{K_{ȴ}}\right),\left(ȴ=1,2\right)$ be any two PFVs. Then, comparisons between PFVs are defined as follows:

i. $S\left(K_{1}\right)>S\left(K_{2}\right)$ implies that $K_{1}>K_{2}$.
ii. $S\left(K_{1}\right)=S\left(K_{2}\right)\;and\;U\left(K_{1}\right)>U\left(K_{2}\right)$ implies that $K_{1}>K_{2}$.
iii. $S\left(K_{1}\right)=S\left(K_{2}\right)\;and\;U\left(K_{1}\right)=U\left(K_{2}\right)$ implies that $K_{1}=K_{2}$.

Definition 10 [58] Suppose $K_{ȴ}=\left(\delta_{K_{ȴ}},{ç}_{K_{ȴ}},\zeta_{K_{ȴ}}\;\right),\left(ȴ=1,2,3,\ldots n\right)$ be a set of PFVs, with associative weight vector $\Xi ={\left({\;\Xi }_{1},\Xi_{2},\ldots \Xi_{n}\right)}^{t}$ of $K_{ȴ}$ such that ${\;\Xi }_{ȴ}>0\text{,}$ and $\sum_{ȴ=1}^{n}\Xi_{ȴ}=1\text{.}$ Then, the picture fuzzy weighted average (PFWA) operator is a mapping $A^{n}\to A$ such that:

$$PFWA_{\Xi }\left(K_{1},K_{2},\ldots K_{n}\right)=\overset{n}{\underset{ȴ=1}{⊕}}\left({\;\Xi }_{ȴ}K_{ȴ}\right)$$

$$=\left(1-\prod_{ȴ=1}^{n}{\left(1-\delta_{K_{ȴ}}\right)}^{\Xi_{ȴ}},\prod_{ȴ=1}^{n}{\left({ç}_{\rho_{ȴ}}\right)}^{\Xi_{ȴ}},\prod_{ȴ=1}^{n}{\left(\zeta_{\rho_{ȴ}}\right)}^{\Xi_{ȴ}}\right)$$

Definition 11 [58] Suppose $K_{ȴ}=\left(\delta_{K_{ȴ}},{ç}_{K_{ȴ}},\zeta_{K_{ȴ}}\;\right),\left(ȴ=1,2,3,\ldots n\right)$ be a set of PFVs, with associative weight vector $\Xi ={\left({\;\Xi }_{1},\Xi_{2},\ldots \Xi_{n}\right)}^{t}$ of $K_{ȴ}$ such that ${\;\Xi }_{ȴ}>0\text{,}$ and $\sum_{ȴ=1}^{n}\Xi_{ȴ}=1\text{.}$ Then, the picture fuzzy weighted geometric (PFWG) operator is a mapping $A^{n}\to A$ such that:

$$PFWG_{\Xi }\left(K_{1},K_{2},\ldots K_{n}\right)=\overset{n}{\underset{ȴ=1}{⨂}}K_{ȴ}^{\Xi_{ȴ}}$$

$$=\left(\prod_{ȴ=1}^{n}{\left(\delta_{K_{ȴ}}\right)}^{\Xi_{ȴ}},1-\prod_{ȴ=1}^{n}{\left(1-\;{ç}_{K_{ȴ}}\right)}^{\Xi_{ȴ}},1-\prod_{ȴ=1}^{n}{\left(1-\;\zeta_{K_{ȴ}}\right)}^{\Xi_{ȴ}}\;\right)$$

Definition 12 [60] Suppose $a_{ȴ}$ be the set of positive real numbers. The power average operator is given by:

$$PA\left(a_{1},a_{2},\ldots ,a_{n}\right)=\frac{\sum_{ȴ=1}^{n}\left(1+A\left(a_{ȴ}\right)\right)a_{ȴ}}{\sum_{ȴ=1}^{n}\left(1+A\left(a_{ȴ}\right)\right)}$$

Where $A\left(a_{ȴ}\right)=\sum_{\begin{array}{c} \;k=1\\ k\neq ȴ\; \end{array}}^{n}Supp\left(a_{ȴ},a_{k}\right)$.

Some necessary characteristics of the support function are expressed as follows:

I. $Supp\left(a_{ȴ},a_{k}\right)\in \left[0,1\right]$.
II. $Supp\left(a_{ȴ},a_{k}\right)=Supp\left(a_{k},a_{ȴ}\right)$.
III. $Supp\left(a_{ȴ},a_{k}\right)\geq Supp\left(a_{s},a_{t}\right),If\left|a_{ȴ}-a_{k}\right|<\left|a_{s},a_{t}\right|$.

Definition 13 [61] Suppose $a_{ȴ}$ be the set of positive real numbers. The power geometric operator is given by:

$$PG\left(a_{1},a_{2},\ldots ,a_{n}\right)=\prod_{ȴ=1}^{n}\left(a_{ȴ}^{\frac{1+A\left(aȴ\;\right)}{\sum_{ȴ=1}^{n}\left(1+A\left(a_{ȴ}\right)\right)}}\right)$$

Where $A\left(a_{ȴ}\right)=\sum_{\begin{array}{c} \;k=1\\ k\neq ȴ\; \end{array}}^{n}Supp\left(a_{ȴ},a_{k}\right)$.

## 3 Aczel Alsina operations based on picture fuzzy information

We studied some appropriate Aczel Alsina aggregation tools by generalizing the theory of triangular norms in light of PF information.

Definition 14 [54] Suppose $K=\left(\delta_{K},{ç}_{K},\zeta_{K}\;\right),K_{1}=\left(\delta_{K_{1}},{ç}_{K_{1}},\zeta_{K_{1}}\;\right)$ and $K_{2}=\left(\delta_{K_{2}},{ç}_{K_{ȴ}},\zeta_{K_{ȴ}}\;\right)$ are three PFVs. Then, some operational laws of Aczel Alsina aggregation tools are given by:

I. $K_{1}⨁K_{2}=\left(\begin{array}{c} 1-e^{-{\left({\left(-\log\left(1-\delta_{K_{1}}\right)\right)}^{\tau }+{\left(-\log\left(1-\delta_{K_{2}}\right)\right)}^{\tau }\right)}^{\frac{1}{\tau }}},\\ e^{-{\left({\left(-\log\left({ç}_{K_{1}}\right)\right)}^{\tau }+{\left(-\log\left({ç}_{K_{2}}\right)\right)}^{\tau }\right)}^{\frac{1}{\tau }}\;},\\ e^{-{\left({\left(-\log\left(\zeta_{B_{1}}\right)\right)}^{\tau }+{\left(-\log\left(\zeta_{B_{2}}\right)\right)}^{\tau }\right)}^{\frac{1}{\tau }}\;} \end{array}\right)$.
II. $K_{1}⨂K_{2}=\left(\begin{array}{c} e^{-{\left({\left(-\log\left(\delta_{K_{1}}\right)\right)}^{\tau }+{\left(-\log\left(\delta_{K_{2}}\right)\right)}^{\tau }\right)}^{\frac{1}{\tau }}},\\ {1-e}^{-{\left({\left(-\log\left(1-{ç}_{K_{1}}\right)\right)}^{\tau }+{\left(-\log\left(1-{ç}_{K_{2}}\right)\right)}^{\tau }\right)}^{\frac{1}{\tau }}\;},\\ 1-e^{-{\left({\left(-\log\left({1-\zeta }_{K_{1}}\right)\right)}^{\tau }+{\left(-\log\left(1-{ç}_{K_{2}}\right)\right)}^{\tau }\right)}^{\frac{1}{\tau }}\;} \end{array}\right)$.
III. $ǟK=\left(\begin{array}{c} 1-e^{-{\left(ǟ{\left(-\log\left(1-\delta_{K}\right)\right)}^{\tau }\right)}^{\frac{1}{\tau }}},\\ e^{-{\left(ǟ{\left(-\log\left({ç}_{K}\right)\right)}^{\tau }\right)}^{\frac{1}{\tau }}},\\ e^{-{\left(ǟ{\left(-\log\left(\zeta_{K}\right)\right)}^{\tau }\right)}^{\frac{1}{\tau }}} \end{array}\right)$.
IV. $K^{ǟ}=\left(\begin{array}{c} e^{-{\left(ǟ{\left(-\log\left(\delta_{K}\right)\right)}^{\tau }\right)}^{\frac{1}{\tau }}},\\ 1-e^{-{\left(ǟ{\left(-\log\left(1-{ç}_{K}\right)\right)}^{\tau }\right)}^{\frac{1}{\tau }}},\\ 1-e^{-{\left(ǟ{\left(-\log\left(1-\zeta_{K}\right)\right)}^{\tau }\right)}^{\frac{1}{\tau }}}\; \end{array}\right)$.

Definition 15 [62] Suppose $K_{ȴ}=\left(\left(\delta_{K_{ȴ}},{ç}_{K_{ȴ}},\zeta_{K_{ȴ}}\;\right),ȴ=1,2,\ldots ,n\right)$ be set of PFVs, with associative weight vectors $\Xi ={\left({\;\Xi }_{1},\Xi_{2},\ldots \Xi_{n}\right)}^{t},\left(ȴ=1,2,\ldots n\right)$ of $K_{ȴ}$ such that ${\;\Xi }_{ȴ}>0$ and $\sum_{ȴ=1}^{n}\Xi_{ȴ}=1$. Then, the picture fuzzy Aczel Alsina weighted average (PFAWA) operator is given by:

$$PFAWA\left(K_{1},K_{2},\ldots ,K_{n}\right)=\overset{n}{\underset{ȴ=1}{⊕}}\left({\;\Xi }_{ȴ}K_{ȴ}\right)$$

$$=\left(\begin{array}{c} 1-e^{-{\left(\sum_{ȴ=1}^{n}\Xi_{ȴ}{\left(-\ln\left(1-\delta_{K_{ȴ}}\right)\right)}^{\tau }\right)}^{\frac{1}{\tau }}},\\ e^{-{\left(\sum_{ȴ=1}^{n}\Xi_{ȴ}{\left(-\log\;{ç}_{K_{ȴ}}\;\right)}^{\tau }\right)}^{\frac{1}{\tau }}},\\ e^{-{\left(\sum_{ȴ=1}^{n}\Xi_{ȴ}{\left(-\log\;\zeta_{K_{ȴ}}\;\right)}^{\tau }\right)}^{\frac{1}{\tau }}} \end{array}\right)$$

Definition 16 [54] Suppose $K_{ȴ}=\left(\left(\delta_{K_{ȴ}},{ç}_{K_{ȴ}},\zeta_{K_{ȴ}}\;\right),ȴ=1,2,\ldots ,n\right)$ be set of PFVs, with associative weight vectors $\Xi ={\left({\;\Xi }_{1},\Xi_{2},\ldots \Xi_{n}\right)}^{t},\left(ȴ=1,2,\ldots n\right)$ of $K_{ȴ}$ such that ${\;\Xi }_{ȴ}>0$ and $\sum_{ȴ=1}^{n}\Xi_{ȴ}=1$. Then, the picture fuzzy Aczel Alsina weighted geometric (PFAWG) operator is given by:

$$PFAWG\left(K_{1},K_{2},\ldots ,K_{n}\right)=\overset{n}{\underset{ȴ=1}{⨂}}K_{ȴ}^{\Xi_{ȴ}}$$

$$=\left(\begin{array}{c} e^{-{\left(\sum_{ȴ=1}^{n}\Xi_{ȴ}{\left(-\ln\left(\delta_{K_{ȴ}}\right)\right)}^{\tau }\right)}^{\frac{1}{\tau }}},\\ 1-e^{-{\left(\sum_{ȴ=1}^{n}\Xi_{ȴ}{\left(-\ln\left(1-{ç}_{K_{ȴ}}\right)\right)}^{\tau }\right)}^{\frac{1}{\tau }}},\\ \;1-e^{-{\left(\sum_{ȴ=1}^{n}\Xi_{ȴ}{\left(-\ln\left(1-\zeta_{K_{ȴ}}\right)\right)}^{\tau }\right)}^{\frac{1}{\tau }}} \end{array}\right)$$

Theorem 1 *Suppose*$K_{ȴ}=\left(\delta_{K_{ȴ}},{ç}_{K_{ȴ}},\zeta_{K_{ȴ}}\;\right),\left(ȴ=1,2,3,\ldots n\right)$*be set of PFVs*. *So*, *we have*:

I. $K_{1}⨁K_{2}=K_{2}⨁K_{1}$.
II. $K_{1}⨂K_{2}=K_{2}⨂K_{1}$.
III. $\tau \left(K_{1}⨁K_{2}\right)=\tau K_{1}⨁\tau K_{2}$.
IV. ${\left(K_{1}⨂K_{2}\right)}^{\tau }={K_{1}}^{\tau }+{K_{2}}^{\tau }$.
V. $\tau_{1}K_{1}⨁\tau_{2}K_{1}=\left(\tau_{1}+\tau_{2}\right)K_{1}$.
VI. ${K_{1}}^{\tau_{1}}⨂{K_{1}}^{\tau_{2}}={K_{1}}^{\left(\tau_{1}+\tau_{2}\right)}$.
VII. ${\left({K_{1}}^{\tau_{1}}\right)}^{\tau_{2}}={K_{1}}^{\tau_{1}\tau_{2}}$.

## 4 Picture fuzzy Aczel Alsina power weighted averaging operators

In this section, we derived some robust mathematical models based on PF information. 10.13039/100014337Furthermore, some associated weight vectors of $K_{ȴ}$ and support values are indicated by $\Xi ={\left({\;\Xi }_{1},\Xi_{2},\ldots \Xi_{n}\right)}^{t},\left(ȴ=1,2,\ldots n\right),\Xi_{ȴ}>0$ and $\sum_{ȴ=1}^{n}\Xi_{ȴ}=1$ and $Ⱥ\left(K_{ȴ}\right)=\sum_{\begin{array}{c} ȴ=1,j=1\\ ȴ\neq j \end{array}}^{n}\Xi_{ȴ}Supp\left(K_{ȴ},K_{j}\;\right),ȴ=1,2,\ldots ,n,j=1,2,\ldots ,n$ throughout this article.

Definition 17 Suppose $K_{ȴ}=\left(\left(\delta_{K_{ȴ}},{ç}_{K_{ȴ}},\zeta_{K_{ȴ}}\;\right),ȴ=1,2,\ldots ,n\right)$ be set of PFVs. The picture fuzzy Aczel Alsina power weighted average (PFAAPWA) operator is expressed in Equation (3), so we have:

(3) $$PFAAPWA\left(K_{1},K_{2},\ldots ,K_{n}\right)=\overset{n}{\underset{ȴ=1}{⨁}}\left(\frac{\Xi_{ȴ}\left(1+Ⱥ\left(K_{ȴ}\right)\right)}{\sum_{ȴ=1}^{n}\Xi_{ȴ}\left(1+Ⱥ\left(K_{ȴ}\right)\right)}K_{ȴ}\right)$$

Theorem 2 Consider $K_{ȴ}=\left(\left(\delta_{K_{ȴ}},{ç}_{K_{ȴ}},\zeta_{K_{ȴ}}\;\right),ȴ=1,2,\ldots ,n\right)$ be set of PFVs. Then, the aggregated value of the PFAAPWA operator is still a PFV and expressed in Equation (4):

(4) $$PFAAPWA\left(K_{1},K_{2},\ldots ,K_{n}\right)=\left(\begin{array}{c} \left(1-e^{-{\left(\sum_{ȴ=1}^{n}\left(Y_{ȴ}\right){\left(-\log\left(1-\delta_{ȴ}\right)\right)}^{\tau }\right)}^{\frac{1}{\tau }}}\right),\\ \left(e^{-{\left(\sum_{ȴ=1}^{n}\left(Y_{ȴ}\right){\left(-\log\left({ç}_{ȴ}\right)\right)}^{\tau }\right)}^{\frac{1}{\tau }}}\right),\\ \left(e^{-{\left(\sum_{ȴ=1}^{n}\left(Y_{ȴ}\right){\left(-\log\left(\zeta_{ȴ}\right)\right)}^{\tau }\right)}^{\frac{1}{\tau }}}\right) \end{array}\right)$$

Where $Y_{ȴ}=\frac{\Xi_{ȴ}\left(1+Ⱥ\left(K_{ȴ}\right)\right)}{\sum_{ȴ=1}^{n}\Xi_{ȴ}\left(1+Ⱥ\left(K_{ȴ}\right)\right)},Ⱥ\left(K_{ȴ}\right)=\sum_{\begin{array}{c} ȴ=1,j=1\\ ȴ\neq j \end{array}}^{n}\Xi_{ȴ}Supp\left(K_{ȴ},K_{j}\;\right),ȴ=1,2,\ldots ,\rho ,j=1,2,\ldots ,n$.

Proof Since $K_{ȴ}=\left(\left(\delta_{K_{ȴ}},{ç}_{K_{ȴ}},\zeta_{K_{ȴ}}\;\right),ȴ=1,2,\ldots ,n\right)$ be set of PFVs, and now we have to prove this theorem by using the following steps for n=2, so we have:

$$PFAAPWA\left(K_{1},K_{2}\right)=\overset{2}{\underset{ȴ=1}{⨁}}\left(\frac{\Xi_{ȴ}\left(1+Ⱥ\left(K_{ȴ}\right)\right)}{\sum_{ȴ=1}^{n}\Xi_{ȴ}\left(1+Ⱥ\left(K_{ȴ}\right)\right)}K_{ȴ}\right)$$

$$\left(\frac{\Xi_{1}\left(1+Ⱥ\left(K_{1}\right)\right)}{\sum_{ȴ=1}^{n}\Xi_{ȴ}\left(1+Ⱥ\left(K_{ȴ}\right)\right)}K_{1}\right)=Y_{1}K_{1}=\left(\begin{array}{c} \left(1-e^{-{\left(\left(Y_{1}\right){\left(-\log\left(1-\delta_{1}\right)\right)}^{\tau }\right)}^{\frac{1}{\tau }}}\right),\\ \left(e^{-{\left(\left(Y_{1}\right){\left(-\log\left({ç}_{1}\right)\right)}^{\tau }\right)}^{\frac{1}{\tau }}}\right),\\ \left(e^{-{\left(\left(Y_{1}\right){\left(-\log\left(\zeta_{1}\right)\right)}^{\tau }\right)}^{\frac{1}{\tau }}}\right) \end{array}\right)$$

$$\left(\frac{\Xi_{2}\left(1+Ⱥ\left(K_{2}\right)\right)}{\sum_{ȴ=1}^{n}\Xi_{ȴ}\left(1+Ⱥ\left(K_{ȴ}\right)\right)}K_{2}\right)=Y_{2}K_{2}=\left(\begin{array}{c} \left(1-e^{-{\left(\left(Y_{2}\right){\left(-\log\left(1-\delta_{2}\right)\right)}^{\tau }\right)}^{\frac{1}{\tau }}}\right),\\ \left(e^{-{\left(\left(Y_{2}\right){\left(-\log\left({ç}_{2}\right)\right)}^{\tau }\right)}^{\frac{1}{\tau }}}\right),\\ \left(e^{-{\left(\left(Y_{2}\right){\left(-\log\left(\zeta_{2}\right)\right)}^{\tau }\right)}^{\frac{1}{\tau }}}\right) \end{array}\right)$$

$$PFAAPWA\left(K_{1},K_{2}\right)=Y_{1}K_{1}⊕Y_{2}K_{2}=\left(\begin{array}{c} \left(\begin{array}{c} \left(1-e^{-{\left(\left(Y_{1}\right){\left(-\log\left(1-\delta_{1}\right)\right)}^{\tau }\right)}^{\frac{1}{\tau }}}\right),\\ \left(e^{-{\left(\left(Y_{1}\right){\left(-\log\left({ç}_{1}\right)\right)}^{\tau }\right)}^{\frac{1}{\tau }}}\right),\\ \left(e^{-{\left(\left(Y_{1}\right){\left(-\log\left(\zeta_{1}\right)\right)}^{\tau }\right)}^{\frac{1}{\tau }}}\right) \end{array}\right)⊕\\ \left(\begin{array}{c} \left(1-e^{-{\left(\left(Y_{2}\right){\left(-\log\left(1-\delta_{2}\right)\right)}^{\tau }\right)}^{\frac{1}{\tau }}}\right),\\ \left(e^{-{\left(\left(Y_{2}\right){\left(-\log\left({ç}_{2}\right)\right)}^{\tau }\right)}^{\frac{1}{\tau }}}\right),\\ \left(e^{-{\left(\left(Y_{2}\right){\left(-\log\left(\zeta_{2}\right)\right)}^{\tau }\right)}^{\frac{1}{\tau }}}\right) \end{array}\right) \end{array}\right)$$

$$=\left(\begin{array}{c} 1-e^{{-\left({\left(Y_{1}\right)\left(-\log\left(1-\delta_{{ǟ}_{1}}\right)\right)}^{\tau }+{\left(Y_{2}\right)\left(-\log\left(1-\delta_{{ǟ}_{2}}\right)\right)}^{\tau }\right)}^{\frac{1}{\tau }}},\\ e^{{-\left({\left(Y_{1}\right)\left(-\log\left({ç}_{{ǟ}_{1}}\right)\right)}^{\tau }+{\left(Y_{2}\right)\left(-\log\left({ç}_{{ǟ}_{2}}\right)\right)}^{\tau }\right)}^{\frac{1}{\tau }}},\\ e^{{-\left({\left(Y_{1}\right)\left(-\log\left(\zeta_{{ǟ}_{1}}\right)\right)}^{\tau }+{\left(Y_{2}\right)\left(-\log\left(\zeta_{{ǟ}_{2}}\right)\right)}^{\tau }\right)}^{\frac{1}{\tau }}} \end{array}\right)$$

$$=\left(\begin{array}{c} 1-e^{-{\left(\sum_{j=1}^{2}\left(Y_{ȴ}\right){\left(\;\log\left(1-\delta_{{ǟ}_{j}}\right)\right)}^{\tau }\right)}^{\frac{1}{\tau }}},\\ e^{-{\left(\sum_{j=1}^{2}\left(Y_{ȴ}\right){\left(-\log\left({ç}_{{ǟ}_{j}}\right)\right)}^{\tau }\right)}^{\frac{1}{\tau }}},\\ e^{-{\left(\sum_{j=1}^{2}\left(Y_{ȴ}\right){\left(-\log\left(\zeta_{{ǟ}_{j}}\right)\right)}^{\tau }\right)}^{\frac{1}{\tau }}} \end{array}\right)$$

Hence, this is true for n=2.

i. Now, suppose that this will be true for n=k. Then, we have:

$$PFAAPWA\left(K_{1},K_{2},\ldots ,K_{n}\right)=\overset{k}{\underset{j=1}{⊕}}Y_{k}{ǟ}_{k}$$

$$=\left(\begin{array}{c} 1-e^{-{\left(\sum_{j=1}^{k}\left(Y_{k}\right){\left(\;\log\left(1-\delta_{{ǟ}_{k}}\right)\right)}^{\tau }\right)}^{\frac{1}{\tau }}},\\ e^{-{\left(\sum_{j=1}^{k}\left(Y_{k}\right){\left(-\log\left({ç}_{{ǟ}_{k}}\right)\right)}^{\tau }\right)}^{\frac{1}{\tau }}},\\ e^{-s{\left(\sum_{j=1}^{k}\left(Y_{k}\right){\left(-\log\left(\zeta_{{ǟ}_{k}}\right)\right)}^{\tau }\right)}^{\frac{1}{\tau }}} \end{array}\right)$$

Now, we have to show that it also holds for j=k+1. We get:

$$PFAAPWA\left(K_{1},K_{2},\ldots ,K_{n}\right)=\overset{k}{\underset{j=1}{⊕}}Y_{k}{ǟ}_{k}⊕Y_{k+1}{ǟ}_{k+1}=\left(\begin{array}{c} \left(\begin{array}{c} 1-e^{-{\left(\sum_{j=1}^{k}\left(Y_{j}\right){\left(\;\log\left(1-\delta_{{ǟ}_{j}}\right)\right)}^{\tau }\right)}^{\frac{1}{\tau }}},\\ e^{-{\left(\sum_{j=1}^{k}\left(Y_{j}\right){\left(-\log\left({ç}_{{ǟ}_{j}}\right)\right)}^{\tau }\right)}^{\frac{1}{\tau }}},\\ e^{-{\left(\sum_{j=1}^{k}\left(Y_{j}\right){\left(-\log\left(\zeta_{{ǟ}_{j}}\right)\right)}^{\tau }\right)}^{\frac{1}{\tau }}} \end{array}\right)⊕\\ \left(\begin{array}{c} 1-e^{-{\left(\left(Y_{k+1}\right){\left(\;\log\left(1-\delta_{k+1}\right)\right)}^{\tau }\right)}^{\frac{1}{\tau }}},\\ e^{-{\left({Y_{k+1}\left(-\log\left({ç}_{k+1}\right)\right)}^{\tau }\right)}^{\frac{1}{\tau }}},\\ e^{-{\left(Y_{k+1}{\left(-\log\left(\zeta_{k+1}\right)\right)}^{\tau }\right)}^{\frac{1}{\tau }}} \end{array}\right) \end{array}\right)$$

$$=\left(\begin{array}{c} 1-e^{-{\left(\sum_{j=1}^{k+1}\left(Y_{j}\right){\left(\;\log\left(1-\delta_{{ǟ}_{j}}\right)\right)}^{\tau }\right)}^{\frac{1}{\tau }}},\\ e^{-{\left(\sum_{j=1}^{k+1}\left(Y_{j}\right){\left(-\log\left({ç}_{{ǟ}_{j}}\right)\right)}^{\tau }\right)}^{\frac{1}{\tau }}},\\ e^{-{\left(\sum_{j=1}^{k+1}\left(Y_{j}\right){\left(-\log\left(\zeta_{{ǟ}_{j}}\right)\right)}^{\tau }\right)}^{\frac{1}{\tau }}} \end{array}\right)$$

Which is true for j=k+1.

A theorem is proved.

Theorem 3 *If all PFVs*$K_{ȴ}=\left(\left(\delta_{K_{ȴ}},{ç}_{K_{ȴ}},\zeta_{K_{ȴ}}\;\right),ȴ=1,2,\ldots ,n\right)$*are equal i*.*e*. $K_{ȴ}=K$. *Then*, *we have*:

$$PFAAPWA_{ȴ}\left(K_{1},K_{2},\ldots ,K_{n}\right)=K$$

Proof *Since*$K_{ȴ}=\left(\delta_{K_{ȴ}},{ç}_{K_{ȴ}},\zeta_{K_{ȴ}}\;\right)=K\left(\;ȴ=1,2,\ldots ,n\right)$*then we have that*:

$$PFAAPWA_{ȴ}\left(K_{1},K_{2},\ldots ,K_{n}\right)=\overset{n}{\underset{ȴ=1}{⊕}}\left(Y_{ȴ}K_{ȴ}\right)$$

$$=\left(\begin{array}{c} 1-e^{-{\left(\sum_{ȴ=1}^{n}Y_{ȴ}{\left(-\log\left(1-\delta_{K_{ȴ}}\right)\right)}^{\tau }\right)}^{\frac{1}{\tau }}},\\ e^{-{\left(\sum_{ȴ=1}^{n}Y_{ȴ}{\left(-\log\left(\;{ç}_{K_{ȴ}}\right)\right)}^{\tau }\right)}^{\frac{1}{\tau }}},\\ e^{-{\left(\sum_{ȴ=1}^{n}Y_{ȴ}{\left(-\log\left(\;\zeta_{K_{ȴ}}\right)\right)}^{\tau }\right)}^{\frac{1}{\tau }}} \end{array}\right)$$

$$=\left(\begin{array}{c} 1-e^{-{\left({\left(-\log\left(1-\delta_{K_{ȴ}}\right)\right)}^{\tau }\right)}^{\frac{1}{\tau }}},\\ e^{-{\left({\left(-\log\;{ç}_{K_{ȴ}}\right)}^{\tau }\right)}^{\frac{1}{\tau }}},\\ e^{-{\left({\left(-\log\;\zeta_{K_{ȴ}}\right)}^{\tau }\right)}^{\frac{1}{\tau }}} \end{array}\right)$$

$$=\left(\delta_{K_{ȴ}},{ç}_{K_{ȴ}}{,\zeta }_{K_{ȴ}}\;\right)=K$$

Thus, $PFAAPWA_{ȴ}\left(K_{1},K_{2},\ldots K_{n}\right)=K$ hold.

Theorem 4 *Suppose*$K_{ȴ}$*and*$K_{ȴ}^{\prime }\left(ȴ=1,2,\ldots ,n\right)$*are two sets of PFVs if*$K_{ȴ}\leq K_{ȴ}^{\prime }$*for all*ȴ. *Then*:

$$PFAAPWA_{ȴ}\left(K_{1},K_{2},\ldots ,K_{n}\right)\leq PFAPWA_{ȴ}\left(K_{1}^{\prime },K_{2}^{\prime },\ldots ,K_{n}^{\prime }\right)$$

Theorem 5 *Suppose*$K_{ȴ}=\left(\left(\delta_{K_{ȴ}},{ç}_{K_{ȴ}},\zeta_{K_{ȴ}}\;\right),ȴ=1,2,\ldots ,n\right)$*be an accumulation of PFVs*. *Suppose*$K^{-}=\min\left(K_{1},K_{2},\ldots ,K_{n}\right)$*and*$K^{+}=\max\left(K_{1},K_{2},\ldots ,K_{n}\right)$. *Then*:

$$K^{-}\leq PFAAPWA_{ȴ}\left(K_{1},K_{2},\ldots n\right)\leq K^{+}$$

Proof Suppose $K_{ȴ}=\left(\left(\delta_{K_{ȴ}},{ç}_{K_{ȴ}},\zeta_{K_{ȴ}}\;\right),ȴ=1,2,\ldots ,n\right)$ be an accumulation of PFVs. Suppose $K^{-}=\min\left(K_{1},K_{2},\ldots ,K_{n}\right)=\left(\delta_{K_{ȴ}}^{-},{ç}_{K_{ȴ}}^{-}{,\zeta }_{K_{ȴ}}^{-}\;\right)$ and $K^{+}=\max\left(K_{1},K_{2},\ldots ,K_{n}\right)=\left(\delta_{K_{ȴ}}^{+},{ç}_{K_{ȴ}}^{+},\zeta_{K_{ȴ}}^{+}\right)$ we have $K^{-}=\min_{ȴ}\left\{\delta_{K_{ȴ}}\right\},K^{-}=\underset{ȴ}{\min\left\{{ç}_{K_{ȴ}}\right\},}K^{-}\max_{ȴ}\left\{\zeta_{K_{ȴ}}\right\},K_{ȴ}^{+}=\max_{ȴ}\left\{\delta_{K_{ȴ}}\right\}$ and $K_{ȴ}^{+}=\max_{ȴ}\left\{{ç}_{K_{ȴ}}\right\},K_{ȴ}^{+}=\min_{ȴ}\left\{\;\zeta_{K_{ȴ}}\right\}$

$$1-{e^{-\left(\sum_{ȴ=1}^{n}Y_{ȴ}{\left(-\log\left(1-\delta_{K_{ȴ}}^{-}\right)\right)}^{\tau }\right)}}^{\frac{1}{\tau }}\leq 1-{e^{-\left(\sum_{ȴ=1}^{n}Y_{ȴ}{\left(-\log\left(1-\delta_{K_{ȴ}}\right)\right)}^{\tau }\right)}}^{\frac{1}{\tau }}\leq 1-{e^{-\left(\sum_{ȴ=1}^{n}Y_{ȴ}{\left(-\log\left(1-\delta_{K_{ȴ}}^{+}\right)\right)}^{\tau }\right)}}^{\frac{1}{\tau }}$$

$${e^{-\left(\sum_{ȴ=1}^{n}Y_{ȴ}{\left(-\log\left(\;{ç}_{K_{ȴ}}^{+}\right)\right)}^{\tau }\right)}}^{\frac{1}{\tau }}\leq {e^{-\left(\sum_{ȴ=1}^{n}Y_{ȴ}{\left(-\log\left(\;{ç}_{K_{ȴ}}\right)\right)}^{\tau }\right)}}^{\frac{1}{\tau }}\leq {e^{-\left(\sum_{ȴ=1}^{n}Y_{ȴ}{\left(-\log\left(\;{ç}_{K_{ȴ}}^{-}\right)\right)}^{\tau }\right)}}^{\frac{1}{\tau }}$$

$${e^{-\left(\sum_{ȴ=1}^{n}Y_{ȴ}{\left(-\log\left(\zeta_{K_{ȴ}}^{+}\right)\right)}^{\tau }\right)}}^{\frac{1}{\tau }}\leq {e^{-\left(\sum_{ȴ=1}^{n}Y_{ȴ}{\left(-\log\left(\;\zeta_{K_{ȴ}}\right)\right)}^{\tau }\right)}}^{\frac{1}{\tau }}\leq {e^{-\left(\sum_{ȴ=1}^{n}Y_{ȴ}{\left(-\log\left(\zeta_{K_{ȴ}}^{-}\right)\right)}^{\tau }\right)}}^{\frac{1}{\tau }}$$

Therefore*,*

$$K^{-}\leq PFAAPWA_{ȴ}\left(K_{1},K_{2},\ldots ,K_{\rho }\right)\leq K^{+}$$

Example 1 Suppose that ${ß}_{1}=\left(0.17,0.51,0.22\right),{ß}_{2}=\left(0.41,0.12,0.27\right),{ß}_{3}=\left(0.15,0.76,0.07\right)$ and ${ß}_{4}=\left(0.55,0.08,0.34\right)$ are four PFVs with corresponding weight vector Ξ=(0.25,0.40,0.20,0.15) of PFVs and τ=3. Now, we integrate given values by using the PFAAPWA operator. So, we have:

Since $Ⱥ\left(K_{ȴ}\right)=\sum_{\begin{array}{c} ȴ=1,j=1\\ ȴ\neq j \end{array}}^{n}\Xi_{ȴ}Supp\left(K_{ȴ},K_{j}\;\right),ȴ=1,2,\ldots ,n,j=1,2,\ldots ,n$ and $Y_{ȴ}=\frac{\Xi_{ȴ}\left(1+Ⱥ\left(K_{ȴ}\right)\right)}{\sum_{ȴ=1}^{n}\Xi_{ȴ}\left(1+Ⱥ\left(K_{ȴ}\right)\right)}$.

$$Y_{1}=0.2578,Y_{2}=0.3794,Y_{3}=0.2018,Y_{4}=0.1610$$

$$PFAAPWA\left(K_{1},K_{2},K_{3},K_{4}\right)=\left(\begin{array}{c} \left(1-e^{-{\left(\sum_{ȴ=1}^{n}\left(Y_{ȴ}\right){\left(-\log\left(1-\delta_{ȴ}\right)\right)}^{3}\right)}^{\frac{1}{3}}}\right),\\ \left(e^{-{\left(\sum_{ȴ=1}^{n}\left(Y_{ȴ}\right){\left(-\log\left({ç}_{ȴ}\right)\right)}^{3}\right)}^{\frac{1}{3}}}\right),\\ \left(e^{-{\left(\sum_{ȴ=1}^{n}\left(Y_{ȴ}\right){\left(-\log\left(\zeta_{ȴ}\right)\right)}^{3}\right)}^{\frac{1}{3}}}\right) \end{array}\right)$$

$$=\left(\begin{array}{c} \left(1-e^{-{\left(\left(0.2578\right){\left(-\log\left(1-0.17\right)\right)}^{3}+\left(0.3794\right){\left(-\log\left(1-0.41\right)\right)}^{3}+\left(0.2018\right){\left(-\log\left(1-0.15\right)\right)}^{3}+\left(0.1610\right){\left(-log\left(1-0.55\right)\right)}^{3}\right)}^{\frac{1}{3}}}\right),\\ \left(e^{-{\left(\left(0.2578\right){\left(-\log\left(0.51\right)\right)}^{3}+\left(0.3794\right){\left(-\log\left(0.12\right)\right)}^{3}+\left(0.2018\right){\left(-\log\left(0.76\right)\right)}^{3}+\left(0.1610\right){\left(-\log\left(0.08\right)\right)}^{3}\right)}^{\frac{1}{3}}}\right),\\ \left(e^{-{\left(\left(0.2578\right){\left(-\log\left(0.22\right)\right)}^{3}+\left(0.3794\right){\left(-\log\left(0.27\right)\right)}^{3}+\left(0.2018\right){\left(-\log\left(0.07\right)\right)}^{3}+\left(0.1610\right){\left(-\log\left(0.34\right)\right)}^{3}\right)}^{\frac{1}{3}}}\right) \end{array}\right)$$

=(0.2020,0.4485,0.4594)

Definition 18 Suppose $K_{ȴ}=\left(\left(\delta_{K_{ȴ}},{ç}_{K_{ȴ}},\zeta_{K_{ȴ}}\;\right),ȴ=1,2,\ldots ,n\right)$ be set of PFVs. Then, the PFAAPOWA operator is defined in Equation (5):

(5) $$PFAAPOWA\left(K_{1},K_{2},\ldots ,K_{n}\right)=\overset{n}{\underset{ȴ=1}{⨁}}\left(\frac{\Xi_{ȴ}\left(1+Ⱥ\left(K_{\vartheta \left(n\right)}\right)\right)}{\sum_{ȴ=1}^{n}\Xi_{ȴ}\left(1+Ⱥ\left(K_{\vartheta \left(n\right)}\right)\right)}K_{\vartheta \left(n\right)}\right)$$

Where $Ⱥ\left(K_{ȴ}\right)=\sum_{\begin{array}{c} ȴ=1,j=1\\ ȴ\neq j \end{array}}^{n}\Xi_{ȴ}Supp\left(K_{ȴ},K_{j}\;\right),ȴ=1,2,\ldots ,n,j=1,2,\ldots ,n$ and (ϑ(1),ϑ(2),…,ϑ(n)) are the permutation of (ȴ=1,2,…,n) including $K_{\vartheta \left(ȴ-1\right)}\geq K_{\vartheta \left(ȴ\right)}$ for all ȴ=1,2,…,ρ.

Theorem 6 Consider $K_{ȴ}=\left(\left(\delta_{K_{ȴ}},{ç}_{K_{ȴ}},\zeta_{K_{ȴ}}\;\right),ȴ=1,2,\ldots ,n\right)$ be set of PFVs. Then, the integrated value of the PFAAPOWA operator is still a PFV, so we can see the Equation (6):

(6) $$PFAAPOWA\left(K_{1},K_{2},\ldots ,K_{n}\right)=\left(\begin{array}{c} \left(1-e^{-{\left(\sum_{ȴ=1}^{n}\left(Y_{ȴ}\right){\left(-\log\left(1-\delta_{\vartheta \left(n\right)}\right)\right)}^{\tau }\right)}^{\frac{1}{\tau }}}\right),\\ \left(e^{-{\left(\sum_{ȴ=1}^{n}\left(Y_{ȴ}\right){\left(-\log\left({ç}_{\vartheta \left(n\right)}\right)\right)}^{\tau }\right)}^{\frac{1}{\tau }}}\right),\\ \left(e^{-{\left(\sum_{ȴ=1}^{n}\left(Y_{ȴ}\right){\left(-\log\left(\zeta_{\vartheta \left(n\right)}\right)\right)}^{\tau }\right)}^{\frac{1}{\tau }}}\right) \end{array}\right)$$

Where $Y_{ȴ}=\frac{\Xi_{ȴ}\left(1+Ⱥ\left(K_{ȴ}\right)\right)}{\sum_{ȴ=1}^{n}\Xi_{ȴ}\left(1+Ⱥ\left(K_{ȴ}\right)\right)},Ⱥ\left(K_{ȴ}\right)=\sum_{\begin{array}{c} ȴ=1,j=1\\ ȴ\neq j \end{array}}^{n}\Xi_{ȴ}\text{Supp}\left(K_{ȴ},K_{j}\;\right),ȴ=1,2,\ldots ,n,j=1,2,\ldots ,n$ and associative weight vectors $\Xi ={\left({\;\Xi }_{1},\Xi_{2},\ldots \Xi_{n}\right)}^{t},\left(ȴ=1,2,\ldots n\right)$ of $K_{ȴ}$ such that ${\;\Xi }_{ȴ}>0$ and $\sum_{ȴ=1}^{n}\Xi_{ȴ}=1$.

Theorem 7 *If all PFVs*$K_{ȴ}=\left(\left(\delta_{K_{ȴ}},{ç}_{K_{ȴ}},\zeta_{K_{ȴ}}\;\right),ȴ=1,2,\ldots ,n\right)$*are equal i*.*e*. $K_{ȴ}=K$. *Then*:

$$PFAAPOWA_{ȴ}\left(K_{1},K_{2},\ldots ,K_{n}\right)=K$$

Theorem 8 *Suppose*$K_{ȴ}$*and*$K_{ȴ}^{\prime }\left(ȴ=1,2,\ldots ,n\right)$*are two sets of PFVs if*$K_{ȴ}\leq K_{ȴ}^{\prime }$. *Then*:

$$PFAAPOWA_{ȴ}\left(K_{1},K_{2},\ldots ,K_{n}\right)\leq PFAPOWA_{ȴ}\left(K_{1}^{\prime },K_{2}^{\prime },\ldots ,K_{n}^{\prime }\right)$$

Theorem 9 *Suppose*$K_{ȴ}=\left(\left(\delta_{K_{ȴ}},{ç}_{K_{ȴ}},\zeta_{K_{ȴ}}\;\right),ȴ=1,2,\ldots ,n\right)$*be an accumulation of PFVs*. *Suppose*$K^{-}=\min\left(K_{1},K_{2},\ldots ,K_{n}\right)$*and*$K^{+}=\max\left(K_{1},K_{2},\ldots ,K_{n}\right)$. *Then*:

$$K^{-}\leq PFAAPOWA_{ȴ}\left(K_{1},K_{2},\ldots n\right)\leq K^{+}$$

## 5 Picture fuzzy Aczel Alsina power weighted geometric operators

In this section, we derived some appropriate methodologies, such as PFAAPWG and PFAAPOWG operators, by exploring the theory of geometric aggregation tools based on PF information.

Definition 19 Consider $K_{ȴ}=\left(\left(\delta_{K_{ȴ}},{ç}_{K_{ȴ}},\zeta_{K_{ȴ}}\;\right),ȴ=1,2,\ldots ,n\right)$ be set of PFVs. Then, the PFAAWG operator is expressed in Equation (7) and we have:

(7) $$PFAAPWG\left(K_{1},K_{2},\ldots ,K_{n}\right)=\overset{\rho }{\underset{ȴ=1}{⨂}}\left(K_{ȴ}^{Y_{ȴ}}\right)$$

Where $Y_{ȴ}=\frac{\Xi_{ȴ}\left(1+Ⱥ\left(K_{ȴ}\right)\right)}{\sum_{ȴ=1}^{n}\Xi_{ȴ}\left(1+Ⱥ\left(K_{ȴ}\right)\right)},Ⱥ\left(K_{ȴ}\right)=\sum_{\begin{array}{c} ȴ=1,j=1\\ ȴ\neq j \end{array}}^{n}\Xi_{ȴ}Supp\left(K_{ȴ},K_{j}\;\right),ȴ=1,2,\ldots ,\rho ,j=1,2,\ldots ,n$.

Theorem 10 Consider $K_{ȴ}=\left(\left(\delta_{K_{ȴ}},{ç}_{K_{ȴ}},\zeta_{K_{ȴ}}\;\right),ȴ=1,2,\ldots ,n\right)$ be set of PFVs. Then, the integrated value of the PFAAPWG operator is still a PFV, so we can see the Equation (8):

(8) $$PFAAPWG\left(K_{1},K_{2},\ldots ,K_{n}\right)=\left(\begin{array}{c} \left(e^{-{\left(\sum_{ȴ=1}^{n}\left(Y_{ȴ}\right){\left(-\log\left(\delta_{ȴ}\right)\right)}^{\tau }\right)}^{\frac{1}{\tau }}}\right),\\ \left(1-e^{-{\left(\sum_{ȴ=1}^{n}\left(Y_{ȴ}\right){\left(-\log\left(1-{ç}_{ȴ}\right)\right)}^{\tau }\right)}^{\frac{1}{\tau }}}\right),\\ \left(1-e^{-{\left(\sum_{ȴ=1}^{n}\left(Y_{ȴ}\right){\left(-\log\left(1-\zeta_{ȴ}\right)\right)}^{\tau }\right)}^{\frac{1}{\tau }}}\right) \end{array}\right)$$

Where $Y_{ȴ}=\frac{\Xi_{ȴ}\left(1+Ⱥ\left(K_{ȴ}\right)\right)}{\sum_{ȴ=1}^{n}\Xi_{ȴ}\left(1+Ⱥ\left(K_{ȴ}\right)\right)},Ⱥ\left(K_{ȴ}\right)=\sum_{\begin{array}{c} ȴ=1,j=1\\ ȴ\neq j \end{array}}^{n}\Xi_{ȴ}\text{Supp}\left(K_{ȴ},K_{j}\;\right),ȴ=1,2,\ldots ,\text{nj}=1,2,\ldots ,n$.

**Prove:** Since $K_{ȴ}=\left(\left(\delta_{K_{ȴ}},{ç}_{K_{ȴ}},\zeta_{K_{ȴ}}\;\right),ȴ=1,2,\ldots ,n\right)$ be set of PFVs. We prove this theorem by using the mathematical technique for n=2. We have:

$$PFAAPWG\left(K_{1},K_{2}\right)=\left(\begin{array}{c} \left(e^{-{\left(\sum_{ȴ=1}^{2}\left(Y_{ȴ}\right){\left(-\log\left(\delta_{ȴ}\right)\right)}^{\tau }\right)}^{\frac{1}{\tau }}}\right),\\ \left(1-e^{-{\left(\sum_{ȴ=1}^{2}\left(Y_{ȴ}\right){\left(-\log\left(1-{ç}_{ȴ}\right)\right)}^{\tau }\right)}^{\frac{1}{\tau }}}\right),\\ \left(1-e^{-{\left(\sum_{ȴ=1}^{2}\left(Y_{ȴ}\right){\left(-\log\left(1-\zeta_{ȴ}\right)\right)}^{\tau }\right)}^{\frac{1}{\tau }}}\right) \end{array}\right)$$

*Where* $Y_{ȴ}=\frac{\Xi_{ȴ}\left(1+Ⱥ\left(K_{ȴ}\right)\right)}{\sum_{ȴ=1}^{n}\Xi_{ȴ}\left(1+Ⱥ\left(K_{ȴ}\right)\right)},Ⱥ\left(K_{ȴ}\right)=\sum_{\begin{array}{c} ȴ=1,j=1\\ ȴ\neq j \end{array}}^{n}\Xi_{ȴ}Supp\left(K_{ȴ},K_{j}\;\right),ȴ=1,2,\ldots ,nj=1,2,\ldots ,n$, *by using the basic operations of Aczel Alsina aggregation tools*.

$$\left(\;K_{1}^{\frac{\Xi_{ȴ}\left(1+Ⱥ\left(K_{ȴ}\right)\right)}{\sum_{ȴ=1}^{n}\Xi_{ȴ}\left(1+Ⱥ\left(K_{ȴ}\right)\right)}}\right)=K_{1}^{Y_{1}}=\left(\begin{array}{c} \left(e^{-{\left(\left(Y_{1}\right){\left(-\log\left(\delta_{1}\right)\right)}^{\tau }\right)}^{\frac{1}{\tau }}}\right),\\ \left(1-e^{-{\left(\left(Y_{1}\right){\left(-\log\left(1-{ç}_{1}\right)\right)}^{\tau }\right)}^{\frac{1}{\tau }}}\right),\\ \left(1-e^{-{\left(\left(Y_{1}\right){\left(-\log\left(1-\zeta_{1}\right)\right)}^{\tau }\right)}^{\frac{1}{\tau }}}\right) \end{array}\right)$$

$$\left(\;K_{2}^{\frac{\Xi_{ȴ}\left(1+Ⱥ\left(K_{ȴ}\right)\right)}{\sum_{ȴ=1}^{n}\Xi_{ȴ}\left(1+Ⱥ\left(K_{ȴ}\right)\right)}}\right)=K_{2}^{Y_{2}}=\left(\begin{array}{c} \left(e^{-{\left(\left(Y_{2}\right){\left(-\log\left(\delta_{2}\right)\right)}^{\tau }\right)}^{\frac{1}{\tau }}}\right),\\ \left(1-e^{-{\left(\left(Y_{2}\right){\left(-\log\left(1-{ç}_{2}\right)\right)}^{\tau }\right)}^{\frac{1}{\tau }}}\right),\\ \left(1-e^{-{\left(\left(Y_{2}\right){\left(-\log\left(1-\zeta_{2}\right)\right)}^{\tau }\right)}^{\frac{1}{\tau }}}\right) \end{array}\right)$$

$$PFAAPWG\left(K_{1},K_{2}\right)=K_{1}^{Y_{1}}⨂K_{2}^{Y_{2}}=\left(\begin{array}{c} \left(\begin{array}{c} \left(e^{-{\left(\left(Y_{1}\right){\left(-\log\left(\delta_{1}\right)\right)}^{\tau }\right)}^{\frac{1}{\tau }}}\right),\\ \left(1-e^{-{\left(\left(Y_{1}\right){\left(-\log\left(1-{ç}_{1}\right)\right)}^{\tau }\right)}^{\frac{1}{\tau }}}\right),\\ \left(1-e^{-{\left(\left(Y_{1}\right){\left(-\log\left(1-\zeta_{1}\right)\right)}^{\tau }\right)}^{\frac{1}{\tau }}}\right) \end{array}\right)⨂\\ \left(\begin{array}{c} \left(e^{-{\left(\left(Y_{2}\right){\left(-\log\left(\delta_{2}\right)\right)}^{\tau }\right)}^{\frac{1}{\tau }}}\right),\\ \left(1-e^{-{\left(\left(Y_{2}\right){\left(-\log\left(1-{ç}_{2}\right)\right)}^{\tau }\right)}^{\frac{1}{\tau }}}\right),\\ \left(1-e^{-{\left(\left(Y_{2}\right){\left(-\log\left(1-\zeta_{2}\right)\right)}^{\tau }\right)}^{\frac{1}{\tau }}}\right) \end{array}\right) \end{array}\right)$$

$$=\left(\begin{array}{c} {e^{-\left(\left(Y_{1}\right){\left(-\log\left(\delta_{1}\right)\right)}^{\tau }+\left(Y_{2}\right){\left(-\log\left(\delta_{2}\right)\right)}^{\tau }\right)}}^{\frac{1}{\tau }}\\ 1-e^{-{\left(\left(Y_{1}\right){\left(-\log\left(1-{ç}_{1}\right)\right)}^{\tau }+\left(Y_{2}\right){\left(-\log\left(1-{ç}_{2}\right)\right)}^{\tau }\right)}^{\frac{1}{\tau }}}\\ 1-e^{-{\left(\left(Y_{1}\right){\left(-\log\left(1-\zeta_{1}\right)\right)}^{\tau }+\left(Y_{2}\right){\left(-\log\left(1-\zeta_{2}\right)\right)}^{\tau }\right)}^{\frac{1}{\tau }}} \end{array}\right)$$

$$=\left(\begin{array}{c} e^{-{\left(\sum_{j=1}^{2}{Y_{ȴ}\left(-\log\left(\delta_{{ǟ}_{j}}\right)\right)}^{\tau }\right)}^{\frac{1}{\tau }}}\\ 1-e^{-{\left(\sum_{j=1}^{2}{Y_{ȴ}\left(-\log\left(1-{ç}_{{ǟ}_{j}}\right)\right)}^{\tau }\right)}^{\frac{1}{\tau }}}\\ 1-e^{-{\left(\sum_{j=1}^{2}Y_{ȴ}{\left(-\log\left(1-\zeta_{{ǟ}_{j}}\right)\right)}^{\tau }\right)}^{\frac{1}{\tau }}} \end{array}\right)$$

Hence, this is true for n=2.

i. *Now*, *suppose that this will be true for*n=k.*Then*, *we have*:

$$PFAAPWG\left(K_{1},K_{2},\ldots ,K_{n}\right)=\overset{k}{\underset{j=1}{⨂}}{ǟ}_{k}^{Y_{k}}$$

$$=\left(\begin{array}{c} e^{-{\left(\sum_{j=1}^{k}Y_{j}{\left(-\log\left(\delta_{{ǟ}_{j}}\right)\right)}^{\tau }\right)}^{\frac{1}{\tau }}},\\ 1-e^{-{\left(\sum_{j=1}^{k}Y_{j}{\left(-\log\left(1-{ç}_{{ǟ}_{j}}\right)\right)}^{\tau }\right)}^{\frac{1}{\tau }}},\\ 1-e^{-{\left(\sum_{j=1}^{k}Y_{j}{\left(-\log\left(1-\zeta_{{ǟ}_{j}}\right)\right)}^{\tau }\right)}^{\frac{1}{\tau }}} \end{array}\right)$$

*Now*, *we have to show that it also holds for*j=k+1. *We get*:

$$PFAAPWG\left(K_{1},K_{2},\ldots ,K_{n}\right)=\overset{k}{\underset{j=1}{⨂}}\left({ǟ}_{j}^{Y_{j}}⨂{ǟ}_{k+1}^{Y_{k+1}}\right)=\left(\begin{array}{c} \left(\begin{array}{c} e^{-{\left(\sum_{j=1}^{k}Y_{j}{\left(-\log\left(\delta_{{ǟ}_{j}}\right)\right)}^{\tau }\right)}^{\frac{1}{\tau }}},\\ 1-e^{-{\left(\sum_{j=1}^{k}Y_{j}{\left(-\log\left(1-{ç}_{{ǟ}_{j}}\right)\right)}^{\tau }\right)}^{\frac{1}{\tau }}},\\ 1-e^{-{\left(\sum_{j=1}^{k}Y_{j}{\left(-\log\left(1-\zeta_{{ǟ}_{j}}\right)\right)}^{\tau }\right)}^{\frac{1}{\tau }}} \end{array}\right)⨂\\ \left(\begin{array}{c} e^{-{\left(Y_{k+1}{\left(\;\log\left(\delta_{k+1}\right)\right)}^{\tau }\right)}^{\frac{1}{\tau }}},\\ {1-e}^{-{\left({Y_{k+1}\left(-\log\left(1-{ç}_{k+1}\right)\right)}^{\tau }\right)}^{\frac{1}{\tau }}},\\ {1-e}^{-{\left(Y_{k+1}{\left(-\log\left({1-\zeta }_{k+1}\right)\right)}^{\tau }\right)}^{\frac{1}{\tau }}} \end{array}\right) \end{array}\right)$$

$$=\left(\begin{array}{c} e^{-{\left(\sum_{j=1}^{k+1}Y_{j}{\left(-\log\left(\delta_{{ǟ}_{j}}\right)\right)}^{\tau }\right)}^{\frac{1}{\tau }}},\\ 1-e^{-{\left(\sum_{j=1}^{k+1}Y_{j}{\left(-\log\left(1-{ç}_{{ǟ}_{j}}\right)\right)}^{\tau }\right)}^{\frac{1}{\tau }}},\\ 1-e^{-{\left(\sum_{j=1}^{k+1}Y_{j}{\left(-\log\left(1-\zeta_{{ǟ}_{j}}\right)\right)}^{\tau }\right)}^{\frac{1}{\tau }}} \end{array}\right)$$

Which is true for j=k+1.

A theorem is proved.

Theorem 11 If all PFVs $K_{ȴ}=\left(\left(\delta_{K_{ȴ}},{ç}_{K_{ȴ}},\zeta_{K_{ȴ}}\;\right),ȴ=1,2,\ldots ,n\right)$ are equal, i.e., $K_{ȴ}=K$. Then:

$$PFAAPWG\left(K_{1},K_{2},\ldots ,K_{n}\right)=K$$

Theorem 12 *Suppose*$K_{ȴ}$*and*$K_{ȴ}^{\prime }\left(ȴ=1,2,\ldots ,n\right)$*are two sets of PFVs if*$K_{ȴ}\leq K_{ȴ}^{\prime }$. *Then*:

$$PFAAPWG\left(K_{1},K_{2},\ldots ,K_{n}\right)\leq PFAPWG\left(K_{1}^{\prime },K_{2}^{\prime },\ldots ,K_{n}^{\prime }\right)$$

Theorem 13 *Suppose*$K_{ȴ}=\left(\left(\delta_{K_{ȴ}},{ç}_{K_{ȴ}},\zeta_{K_{ȴ}}\;\right),ȴ=1,2,\ldots ,n\right)$*be an accumulation of PFVs*. *Suppose*$K^{-}=\min\left(K_{1},K_{2},\ldots ,K_{n}\right)$*and*$K^{+}=\max\left(K_{1},K_{2},\ldots ,K_{n}\right)$. *Then*:

$$K^{-}\leq PFAAPWG\left(K_{1},K_{2},\ldots n\right)\leq K^{+}$$

Example 2 *Suppose that*${ß}_{1}=\left(0.09,0.19,0.37\right),{ß}_{2}=\left(0.18,0.52,0.26\right),{ß}_{3}=\left(0.45,0.04,0.34\right)$*and*${ß}_{4}=\left(0.31,0.17,0.42\right)$*are four PFVs with corresponding weight vector*Ξ=(0.35,0.30,0.20,0.15)*of PFVs and*τ=3. *Now*, *we integrate given values by using the PFAAPWG operator*. *So*, *we have*:

Since $Ⱥ\left(K_{ȴ}\right)=\sum_{\begin{array}{c} ȴ=1,j=1\\ ȴ\neq j \end{array}}^{n}\Xi_{ȴ}\text{Supp}\left(K_{ȴ},K_{j}\;\right),ȴ=1,2,\ldots ,n,j=1,2,\ldots ,n$ and $Y_{ȴ}=\frac{\Xi_{ȴ}\left(1+Ⱥ\left(K_{ȴ}\right)\right)}{\sum_{ȴ=1}^{n}\Xi_{ȴ}\left(1+Ⱥ\left(K_{ȴ}\right)\right)}$.

$$Y_{1}=0.3398,Y_{2}=0.2920,Y_{3}=0.2054,Y_{4}=0.1628$$

$$PFAAPWG\left(K_{1},K_{2},K_{3},K_{4}\right)=\left(\begin{array}{c} \left(e^{-{\left(\sum_{ȴ=1}^{n}\left(Y_{ȴ}\right){\left(-\log\left(\delta_{ȴ}\right)\right)}^{3}\right)}^{\frac{1}{3}}}\right),\\ \left(1-e^{-{\left(\sum_{ȴ=1}^{n}\left(Y_{ȴ}\right){\left(-\log\left(1-{ç}_{ȴ}\right)\right)}^{3}\right)}^{\frac{1}{3}}}\right),\\ \left(1-e^{-{\left(\sum_{ȴ=1}^{n}\left(Y_{ȴ}\right){\left(-\log\left(1-\zeta_{ȴ}\right)\right)}^{3}\right)}^{\frac{1}{3}}}\right) \end{array}\right)$$

$$=\left(\begin{array}{c} \left(e^{-{\left(\left(0.3398\right){\left(-\log\left(0.09\right)\right)}^{3}+\left(0.2920\right){\left(-\log\left(0.18\right)\right)}^{3}+\left(0.2054\right){\left(-\log\left(0.45\right)\right)}^{3}+\left(0.1628\right){\left(-\log\left(0.31\right)\right)}^{3}\right)}^{\frac{1}{3}}}\right),\\ \left(1-e^{-{\left(\left(0.3398\right){\left(-\log\left(1-0.19\right)\right)}^{3}+\left(0.2920\right){\left(-\log\left(1-0.52\right)\right)}^{3}+\left(0.2054\right){\left(-\log\left(1-0.04\right)\right)}^{3}+\left(0.1628\right){\left(-\log\left(1-0.17\right)\right)}^{3}\right)}^{\frac{1}{3}}}\right),\\ \left(1-e^{-{\left(\left(0.3398\right){\left(-\log\left(1-0.37\right)\right)}^{3}+\left(0.2920\right){\left(-\log\left(1-0.26\right)\right)}^{3}+\left(0.2054\right){\left(-\log\left(1-0.34\right)\right)}^{3}+\left(0.1628\right){\left(-\log\left(1-0.42\right)\right)}^{3}\right)}^{\frac{1}{3}}}\right) \end{array}\right)$$

=(0.4834,0.1315,0.1664)

Definition 20 Suppose $K_{ȴ}=\left(\left(\delta_{K_{ȴ}},{ç}_{K_{ȴ}},\zeta_{K_{ȴ}}\;\right),ȴ=1,2,\ldots ,n\right)$ be set of PFVs. Then, the PFAAPOWG operator is defined in Equation (9):

(9) $$PFAAPOWG\left(K_{1},K_{2},\ldots ,K_{n}\right)=\overset{n}{\underset{ȴ=1}{⨂}}\left(K_{\vartheta \left(n\right)}^{\frac{\Xi_{ȴ}\left(1+Ⱥ\left(K_{\vartheta \left(n\right)}\right)\right)}{\sum_{ȴ=1}^{n}\Xi_{ȴ}\left(1+Ⱥ\left(K_{\vartheta \left(n\right)}\right)\right)}}\right)$$

Where $Y_{ȴ}=\frac{\Xi_{ȴ}\left(1+Ⱥ\left(K_{\vartheta \left(n\right)}\right)\right)}{\sum_{ȴ=1}^{n}\Xi_{ȴ}\left(1+Ⱥ\left(K_{\vartheta \left(n\right)}\right)\right)},Ⱥ\left(K_{ȴ}\right)=\sum_{\begin{array}{c} ȴ=1,j=1\\ ȴ\neq j \end{array}}^{n}\Xi_{ȴ}\text{Supp}\left(K_{ȴ},K_{j}\;\right),ȴ=1,2,\ldots ,n,j=1,2,\ldots ,n$ and (ϑ(1),ϑ(2),…,ϑ(n)) are the permutation of (ȴ=1,2,…,n) including $K_{\vartheta \left(ȴ-1\right)}\geq K_{\vartheta \left(ȴ\right)}$ for all ȴ=1,2,…,ρ.

Theorem 14 Consider $K_{ȴ}=\left(\left(\delta_{K_{ȴ}},{ç}_{K_{ȴ}},\zeta_{K_{ȴ}}\;\right),ȴ=1,2,\ldots ,n\right)$ be set of PFVs. Then, the integrated value of the PFAAPOWG operator is still a PFV and we can see the Equation (10):

(10) $$PFAAPOWG\left(K_{1},K_{2},\ldots ,K_{n}\right)=\left(\begin{array}{c} \left(e^{-{\left(\sum_{ȴ=1}^{n}\left(Y_{ȴ}\right){\left(-\log\left(\delta_{\vartheta \left(n\right)}\right)\right)}^{\tau }\right)}^{\frac{1}{\tau }}}\right),\\ \left(1-e^{-{\left(\sum_{ȴ=1}^{n}\left(Y_{ȴ}\right){\left(-\log\left(1-{ç}_{\vartheta \left(n\right)}\right)\right)}^{\tau }\right)}^{\frac{1}{\tau }}}\right),\\ \left(1-e^{-{\left(\sum_{ȴ=1}^{n}\left(Y_{ȴ}\right){\left(-\log\left(1-\zeta_{\vartheta \left(n\right)}\right)\right)}^{\tau }\right)}^{\frac{1}{\tau }}}\right) \end{array}\right)$$

Where $Y_{ȴ}=\frac{\Xi_{ȴ}\left(1+Ⱥ\left(K_{\vartheta \left(n\right)}\right)\right)}{\sum_{ȴ=1}^{n}\Xi_{ȴ}\left(1+Ⱥ\left(K_{\vartheta \left(n\right)}\right)\right)},Ⱥ\left(K_{ȴ}\right)=\sum_{\begin{array}{c} ȴ=1,j=1\\ ȴ\neq j \end{array}}^{n}\Xi_{ȴ}\text{Supp}\left(K_{ȴ},K_{j}\;\right),ȴ=1,2,\ldots ,n,j=1,2,\ldots ,n$ and (ϑ(1),ϑ(2),…,ϑ(n)) are the permutation of (ȴ=1,2,…,n) including $K_{\vartheta \left(ȴ-1\right)}\geq K_{\vartheta \left(ȴ\right)}$ for all ȴ=1,2,…,ρ.

Theorem 15 *If all PFVs*$K_{ȴ}=\left(\left(\delta_{K_{ȴ}},{ç}_{K_{ȴ}},\zeta_{K_{ȴ}}\;\right),ȴ=1,2,\ldots ,n\right)$*are equal*, *i*.*e*., $K_{ȴ}=K$. *Then*:

$$PFAAPOWG\left(K_{1},K_{2},\ldots ,K_{n}\right)=K$$

Theorem 16 *Suppose*$K_{ȴ}$*and*$K_{ȴ}^{\prime }\left(ȴ=1,2,\ldots ,n\right)$*are two sets of PFVs*, *if*$K_{ȴ}\leq K_{ȴ}^{\prime }$. *Then*:

$$PFAAPOWG\left(K_{1},K_{2},\ldots ,K_{n}\right)\leq PFAPOWG\left(K_{1}^{\prime },K_{2}^{\prime },\ldots ,K_{n}^{\prime }\right)$$

Theorem 17 *Suppose*$K_{ȴ}=\left(\left(\delta_{K_{ȴ}},{ç}_{K_{ȴ}},\zeta_{K_{ȴ}}\;\right),ȴ=1,2,\ldots ,n\right)$*be an accumulation of PFVs*. *Suppose*$K^{-}=\min\left(K_{1},K_{2},\ldots ,K_{n}\right)$*and*$K^{+}=\max\left(K_{1},K_{2},\ldots ,K_{n}\right)$. *Then*:

$$K^{-}\leq PFAAPOWG\left(K_{1},K_{2},\ldots n\right)\leq K^{+}$$

## 6 Assessment of a MAGDM approach based on picture fuzzy information

In this section, we will evaluate the MAGDM problem to solve the given information of PFVs by utilizing our derived approaches of the PFAAPWA and PFAAPWG operators. Suppose that a collection of alternatives is denoted as $\varpi =\left\{\varpi_{1},\varpi_{2},\ldots ,\varpi_{m}\right\}\;ȴ=1,2,\ldots ,m$ and the set of attributes denoted as $\Phi =\left\{\Phi_{1},\Phi_{2},\ldots ,\Phi_{\rho }\right\},\left(j=1,2,\ldots \rho \right)$ with the given degree of attributes by the decision maker $\Xi ={\left({\;\Xi }_{1},\Xi_{2},\ldots \Xi_{n}\right)}^{t},\left(j=1,2,\ldots \rho \right)$ of $K_{ȴ}$ such that ${\;\Xi }_{j}>0$ and $\sum_{j=1}^{\rho }\Xi_{j}=1$ An expert is denoted by $\varphi_{l}=\left(\varphi_{1},\varphi_{2},\ldots ,\varphi_{r}\right),\left(l=1,2,\ldots ,r\right)$. Which is utilized to assess PF information associative weights vector $\psi_{l}={\left({\;\psi }_{1},\psi_{2},\ldots \psi_{r}\right)}^{t},\left(l=1,2,\ldots ,r\right)$ such that ${\;\psi }_{l}>0$ and $\sum_{l=1}^{r}\psi_{l}=1$. The experts evaluate PF information in the form of $K_{ȴj}=\left(\delta_{ȴj},{ç}_{ȴj},\zeta_{ȴj}\right)$ and construct a decision matrix $S={\left(K_{ȴj}\right)}_{m\times \rho }$. Each PFVs $K_{ȴj}=\left(\delta_{ȴj},{ç}_{ȴj},\zeta_{ȴj}\right)$ contains $\delta_{ȴj}\in \left[0,1\right],{ç}_{ȴj}\in \left[0,1\right],\zeta_{ȴj}\in \left[0,1\right]$ and satisfy such condition $0\leq \delta_{ȴj}+\gamma_{ȴj}+\zeta_{ȴj}\leq 1$. To evaluate the proposed MAGDM problem, we produced an algorithm based on PF information.

### 6.1 Algorithm

**Step 1:** PF information enclosed in different decision matrices by the decision maker.

$$S^{l}={\left(K_{ȴj}^{l}\right)}_{m\times \rho }=\left(\begin{array}{ccc} \begin{array}{c} \left(\delta_{K_{11}},{ç}_{K_{11}},\zeta_{K_{11}}\right)\\ \left(\delta_{K_{21}},{ç}_{K_{21}},\zeta_{K_{21}}\right)\\ \begin{array}{c} \vdots \\ \left(\delta_{K_{m1}},{ç}_{K_{m1}},\zeta_{K_{m1}}\right) \end{array} \end{array} & \begin{array}{c} \left(\delta_{K_{12}},{ç}_{K_{12}},\zeta_{K_{12}}\right)\\ \left(\delta_{K_{22}},{ç}_{K_{22}},\zeta_{K_{22}}\right)\\ \begin{array}{c} \vdots \\ \left(\delta_{K_{m2}},{ç}_{K_{m2}},\zeta_{K_{m2}}\right) \end{array} \end{array} & \begin{array}{cc} \begin{array}{c} \cdots \\ \cdots \\ \begin{array}{c} \ddots \\ \cdots \end{array} \end{array} & \begin{array}{c} \left(\delta_{K_{1\rho }},{ç}_{K_{1\rho }},\zeta_{K_{1\rho }}\right)\\ \left(\delta_{K_{2\rho }},{ç}_{K_{2\rho }},\zeta_{K_{2\rho }}\right)\\ \begin{array}{c} \vdots \\ \left(\delta_{K_{m\rho }},{ç}_{K_{m\rho }},\zeta_{K_{m\rho }}\right) \end{array} \end{array} \end{array} \end{array}\right)$$

**Step 2:** Analysis of the categories of a set of attributes. If more than one type of attribute exists, then we perform the following task to normalize decision matrices.

$$\bar{S^{l}}={\left({\bar{K^{l}}}_{\;ȴj}\right)}_{m\times \rho }=\left\{ \begin{array}{c} K_{ȴj\;}=\left(\delta_{ȴj}^{l},{ç}_{ȴj}^{l},\zeta_{ȴj}^{l}\right)\;for\;benefit\;attribute\;j_{ȴ}\\ {K^{l}}_{ȴj}^{\prime }==\left(\zeta_{ȴj}^{l},{ç}_{ȴj}^{l},\delta_{ȴj}^{l}\;\right)\;for\;cost\;attribute\;j_{ȴ} \end{array}\right.$$

Where ${\left(K_{ȴj}^{l}\right)}^{\prime }$ is the complement of $K_{ȴj}^{l}$ so ${\left(K_{ȴj}^{l}\right)}^{\prime }=\left(\zeta_{ȴj}^{l},{ç}_{ȴj}^{l},\delta_{ȴj}^{l}\right)$ all the different l=1,2,…,r and (ȴ=1,2,…,m)**.** Thus, the decision matrices $S^{l}={\left(K_{ȴj}^{l}\right)}_{m\times \rho }$ can be written as $\bar{S^{l}}={\left({\bar{K^{l}}}_{ȴj}\right)}_{m\times \rho }$.

**Step 3:** Compute supports by using the following Equation (11):

(11) $$Supp\left(K_{ȴj}^{l},K_{ȴs}^{e}\;\right)=1-D\left(K_{ȴj}^{l},K_{ȴs}^{e}\right),\left(ȴ=1,2,\ldots ,m\right),\left(j,s=1,2,\ldots ,\rho \right),\left(l,e=1,2,\ldots ,r\right)$$

$$D\left(K_{ȴj}^{l},K_{ȴs}^{e}\right)=\frac{1}{3}\left(\left|\delta_{ȴj}^{l}-\delta_{ȴs}^{e}\right|+\left|{ç}_{ȴj}^{l}-{ç}_{ȴs}^{e}\right|+\left|\zeta_{ȴj}^{l}-\zeta_{ȴs}^{e}\right|\right)$$

**Step 4:** Compute supports by using the following Equation (12):

(12) $${Ⱥ}^{l}\left(K_{ȴj}^{l}\right)=\sum_{\begin{array}{c} ȴ=1,j=1\\ j\neq s \end{array}}^{\rho }{\;\psi }_{j}Supp\left(K_{ȴj}^{l},K_{ȴs}^{e}\right)$$

Where ${Ⱥ}^{l}\left(K_{ȴj}\right)=\sum_{\begin{array}{c} ȴ=1,j=1\\ ȴ\neq j \end{array}}^{n}{\;\psi }_{l}Supp\left(K_{ȴj}^{l},K_{ȴs}^{e}\;\right),\left(ȴ=1,2,\ldots ,m\right),\left(j,s=1,2,\ldots ,\rho \right),\left(l,e=1,2,\ldots ,r\right)$.

**Step 5:** Aggregate degree of weighted support by using Equation (13):

(13) $$Y_{ȴj}^{l}=\frac{{\;\psi }_{l}\left(1+Ⱥ\left(K_{ȴj}^{l}\right)\right)}{\sum_{l=1}^{r}{\;\psi }_{l}\left(1+Ⱥ\left(K_{ȴj}^{l}\right)\right)}$$

**Step 6:** To accumulate given picture fuzzy information by applying Equation (14) and Equation (15):

(14) $$PFAAPWA\left(\;K_{ȴj}^{1},K_{ȴj}^{2},\ldots ,K_{ȴj}^{l}\right)=\left(\begin{array}{c} \left(1-e^{-{\left(\sum_{ȴ=1}^{n}\left(Y_{ȴ}\right){\left(-\log\left(1-\delta_{ȴ}\right)\right)}^{\tau }\right)}^{\frac{1}{\tau }}}\right),\\ \left(e^{-{\left(\sum_{ȴ=1}^{n}\left(Y_{ȴ}\right){\left(-\log\left({ç}_{ȴ}\right)\right)}^{\tau }\right)}^{\frac{1}{\tau }}}\right),\\ \left(e^{-{\left(\sum_{ȴ=1}^{n}\left(Y_{ȴ}\right){\left(-\log\left(\zeta_{ȴ}\right)\right)}^{\tau }\right)}^{\frac{1}{\tau }}}\right) \end{array}\right)$$,

and

(15) $$PFAAPWG\left(\;K_{ȴj}^{1},K_{ȴj}^{2},\ldots ,K_{ȴj}^{l}\right)=\left(\begin{array}{c} \left(e^{-{\left(\sum_{ȴ=1}^{n}\left(Y_{ȴ}\right){\left(-\log\left(\delta_{ȴ}\right)\right)}^{\tau }\right)}^{\frac{1}{\tau }}}\right),\\ \left(1-e^{-{\left(\sum_{ȴ=1}^{n}\left(Y_{ȴ}\right){\left(-\log\left(1-{ç}_{ȴ}\right)\right)}^{\tau }\right)}^{\frac{1}{\tau }}}\right),\\ \left(1-e^{-{\left(\sum_{ȴ=1}^{n}\left(Y_{ȴ}\right){\left(-\log\left(1-\zeta_{ȴ}\right)\right)}^{\tau }\right)}^{\frac{1}{\tau }}}\right) \end{array}\right)$$

**Step 7:** Again, compute the supports by using the following expression of Equation (16):

(16) $$Supp\left(K_{ȴj},K_{ȴs}\;\right)=1-D\left(K_{ȴj},K_{ȴs}\right),ȴ=1,2,\ldots ,m,j=1,2,\ldots ,\rho \text{,}$$

**Step 8:** Compute additional supports by using Equation (17):

(17) $$Ⱥ\left(K_{ȴj}\right)=\sum_{\begin{array}{c} ȴ=1,j=1\\ ȴ\neq j \end{array}}^{\rho }{\;\Xi }_{ȴ}Supp\left(K_{ȴj},K_{ȴs}\;\right),\left(ȴ=1,2,\ldots ,m\right),\left(\;j,s=1,2,\ldots ,\rho \right)$$

**Step 9:** Degree of additional weighted support by using Equation (18):

(18) $$Y_{ȴj}=\frac{{\;\Xi }_{ȴ}\left(1+Ⱥ\left(K_{ȴj}\right)\right)}{\sum_{ȴ=1}^{n}{\;\Xi }_{ȴ}\left(1+Ⱥ\left(K_{ȴj}\right)\right)},\left(ȴ=1,2,\ldots ,m\right)$$

**Step 10:** Evaluate the given information by using the derived approaches PFAAWA and PFAAWG operators of Equation (19) and Equation (20):

(19) $$PFAAPWA\left(\;K_{ȴ2},K_{ȴ2},\ldots ,K_{ȴj}\right)=\left(\begin{array}{c} \left(1-e^{-{\left(\sum_{ȴ=1}^{n}\left(Y_{ȴ}\right){\left(-\log\left(1-\delta_{ȴ}\right)\right)}^{\tau }\right)}^{\frac{1}{\tau }}}\right),\\ \left(e^{-{\left(\sum_{ȴ=1}^{n}\left(Y_{ȴ}\right){\left(-\log\left({ç}_{ȴ}\right)\right)}^{\tau }\right)}^{\frac{1}{\tau }}}\right),\\ \left(e^{-{\left(\sum_{ȴ=1}^{n}\left(Y_{ȴ}\right){\left(-\log\left(\zeta_{ȴ}\right)\right)}^{\tau }\right)}^{\frac{1}{\tau }}}\right) \end{array}\right)$$,

and

(20) $$PFAAPWG\left(\;K_{ȴ2},K_{ȴ2},\ldots ,K_{ȴj}\right)=\left(\begin{array}{c} \left(e^{-{\left(\sum_{ȴ=1}^{n}\left(Y_{ȴ}\right){\left(-\log\left(\delta_{ȴ}\right)\right)}^{\tau }\right)}^{\frac{1}{\tau }}}\right),\\ \left(1-e^{-{\left(\sum_{ȴ=1}^{n}\left(Y_{ȴ}\right){\left(-\log\left(1-{ç}_{ȴ}\right)\right)}^{\tau }\right)}^{\frac{1}{\tau }}}\right),\\ \left(1-e^{-{\left(\sum_{ȴ=1}^{n}\left(Y_{ȴ}\right){\left(-\log\left(1-\zeta_{ȴ}\right)\right)}^{\tau }\right)}^{\frac{1}{\tau }}}\right) \end{array}\right)$$

**Step 11:** Determined score values by using the consequences of PFAAPWA and PFAAPWG operators. We cannot investigate an appropriate optimal option if the results of score values are identical. The accuracy function is more applicable to handling such situations and provides some realistic options under-discussed criteria.
**Step 12:** To assess a suitable alternative, re-arrange all the score values of an individual and alternatives.

### 6.2 Numerical example

In this practical case study, we evaluate information about five different air conditioner inverters $k=\left\{{ℷ}_{1},{ℷ}_{2},{ℷ}_{3},{ℷ}_{4},{ℷ}_{5}\right\}$. For the assessment of information, there are three experts who are hired $L=\left\{L_{1},L_{2},L_{3}\right\}$ with some prominent degrees of weights (0.35,0.25,0.40). The administrative authority buys new air conditioner investors based on certain characteristics such as $E_{1}$ remote control function, $E_{2}$ quality and cooling capacity, $E_{3}$ consuming rate of energy, $E_{4}$ airflow ability and dehumidifier. We applied our derived methodologies to evaluate the information of air conditioner investors based on assigning degrees of characteristics such as (0.35,0.30,0.25,0.10). Decision matrices 1–3 contain information on air conditioners in the form of PFVs. We evaluate given decision matrices 1–3 by considering the above-defined algorithm.

### 6.3 The Procedure of decision-making approach

**Step 1:** Assemblage information of PF information by the experts in the following Table 1, Table 2, Table 3.

**Table 1:** Contains information about PFVs in Decision Matrix 1.

	${ℶ}_{1}$	${ℶ}_{2}$	${ℶ}_{3}$	${ℶ}_{4}$
${ℷ}_{1}$	(0.47,0.35,0.14)	(0.45,0.15,0.38)	(0.40,0.30,0.10)	(0.11,0.19,0.55)
${ℷ}_{2}$	(0.26,0.63,0.09)	(0.42,0.18,0.32)	(0.25,0.35,0.39)	(0.15,0.25,0.55)
${ℷ}_{3}$	(0.16,0.43,0.32)	(0.55,0.10,0.30)	(0.15,0.35,0.20)	(0.19,0.29,0.49)
${ℷ}_{4}$	(0.56,0.09,0.18)	(0.46,0.30,0.15)	(0.80,0.06,0.10)	(0.33,0.53,0.13)
${ℷ}_{5}$	(0.45,0.32,0.15)	(0.33,0.42,0.11)	(0.30,0.55,0.10)	(0.23,0.43,0.13)

**Table 2:** Contains information about PFVs in Decision Matrix 2.

	${ℶ}_{1}$	${ℶ}_{2}$	${ℶ}_{3}$	${ℶ}_{4}$
${ℷ}_{1}$	(0.18,0.25,0.19)	(0.40,0.40,0.19)	(0.27,0.37,0.09)	(0.66,0.21,0.08)
${ℷ}_{2}$	(0.19,0.29,0.35)	(0.51,0.11,0.13)	(0.27,0.37,0.15)	(0.64,0.20,0.09)
${ℷ}_{3}$	(0.22,0.32,0.42)	(0.61,0.09,0.12)	(0.25,0.35,0.15)	(0.55,0.12,0.18)
${ℷ}_{4}$	(0.21,0.31,0.41)	(0.71,0.05,0.09)	(0.23,0.32,0.16)	(0.44,0.30,0.07)
${ℷ}_{5}$	(0.20,0.30,0.40)	(0.51,0.10,0.08)	(0.20,0.30,0.10)	(0.80,0.11,0.05)

**Table 3:** Contains information about PFVs in Decision Matrix 3.

	${ℶ}_{1}$	${ℶ}_{2}$	${ℶ}_{3}$	${ℶ}_{4}$
${ℷ}_{1}$	(0.55,0.18,0.12)	(0.16,0.47,0.35)	(0.14,0.45,0.09)	(0.33,0.41,0.15)
${ℷ}_{2}$	(0.44,0.35,0.19)	(0.18,0.21,0.58)	(0.09,0.33,0.31)	(0.36,0.23,0.22)
${ℷ}_{3}$	(0.35,0.45,0.09)	(0.21,0.16,0.43)	(0.32,0.41,0.16)	(0.32,0.22,0.28)
${ℷ}_{4}$	(0.16,0.19,0.31)	(0.09,0.56,0.09)	(0.18,0.38,0.21)	(0.19,0.32,0.41)
${ℷ}_{5}$	(0.19,0.21,0.35)	(0.15,0.45,0.32)	(0.15,0.18,0.19)	(0.17,0.44,0.21)
**Step 2:** Only beneficial attributes are exposed in the above-discussed experimental case study, so there is no need to change standard decision matrices into normalized matrices.

**Step 3:** Compute the degree of weighted supports by using Equations (11), (12), (13) and aggregated results are shown as follows:

$$Y^{1}=Y_{ȴj}^{1}=\left(\begin{array}{cc} \begin{array}{ccc} 0.3516 & 0.3474 & 0.3471\\ 0.3471 & 0.3561 & 0.3506\\ \begin{array}{c} 0.3522\\ 0.3426\\ 0.3431 \end{array} & \begin{array}{c} 0.3544\\ 0.3568\\ 0.3565 \end{array} & \begin{array}{c} 0.3478\\ 0.3363\\ 0.3453 \end{array} \end{array} & \begin{array}{c} 0.3442\\ 0.3447\\ \begin{array}{c} 0.3469\\ 0.3501\\ 0.3591 \end{array} \end{array} \end{array}\right)$$

$$Y^{2}=Y_{ȴj}^{2}=\left(\begin{array}{cc} \begin{array}{ccc} 0.2596 & 0.2654 & 0.2670\\ 0.2625 & 0.2613 & 0.2622\\ \begin{array}{c} 0.2630\\ 0.2645\\ 0.2673 \end{array} & \begin{array}{c} 0.2627\\ 0.2587\\ 0.2566 \end{array} & \begin{array}{c} 0.2658\\ 0.2718\\ 0.2683 \end{array} \end{array} & \begin{array}{c} 0.2623\\ 0.2598\\ \begin{array}{c} 0.2590\\ 0.2647\\ 0.2463 \end{array} \end{array} \end{array}\right)$$

$$Y^{3}=Y_{ȴj}^{3}=\left(\begin{array}{cc} \begin{array}{ccc} 0.3888 & 0.3872 & 0.3859\\ 0.3904 & 0.3826 & 0.3871\\ \begin{array}{c} 0.3848\\ 0.3928\\ 0.3896 \end{array} & \begin{array}{c} 0.3829\\ 0.3845\\ 0.3869 \end{array} & \begin{array}{c} 0.3864\\ 0.3919\\ 0.3864 \end{array} \end{array} & \begin{array}{c} 0.3935\\ 0.3955\\ \begin{array}{c} 0.3941\\ 0.3852\\ 0.3945 \end{array} \end{array} \end{array}\right)$$

**Step 4:** Evaluate the given information on PFVs by using Equation (14) and Equation (15), obtaining results in Table 4 and Table 5, respectively.

**Table 4:** Shows the accumulated results for the PFAPWA Operator.

	${ℶ}_{1}$	${ℶ}_{2}$	${ℶ}_{3}$	${ℶ}_{4}$
${ℷ}_{1}$	(0.2244,0.5455,0.4294)	(0.1634,0.5952,0.5981)	(0.1296,0.6501,0.3571)	(0.1884,0.5608,0.4959)
${ℷ}_{2}$	(0.1544,0.6779,0.4657)	(0.1798,0.4774,0.6160)	(0.0911,0.6316,0.5733)	(0.1945,0.5265,0.5373)
${ℷ}_{3}$	(0.1193,0.6752,0.5088)	(0.2363,0.3930,0.5669)	(0.1153,0.6509,0.4632)	(0.1710,0.5045,0.5956)
${ℷ}_{4}$	(0.1639,0.4602,0.5727)	(0.2214,0.5380,0.3804)	(0.2589,0.4916,0.4412)	(0.1502,0.6534,0.4653)
${ℷ}_{5}$	(0.1398,0.5635,0.5674)	(0.1553,0.5915,0.4428)	(0.1013,0.5959,0.4097)	(0.2172,0.6014,0.4041)

**Table 5:** Shows the accumulated results for the PFAPWG Operator.

	${ℶ}_{1}$	${ℶ}_{2}$	${ℶ}_{3}$	${ℶ}_{4}$
${ℷ}_{1}$	(0.6639,0.1234,0.0661)	(0.5859,0.1727,0.1559)	(0.5649,0.1874,0.0417)	(0.5649,0.1371,0.1468)
${ℷ}_{2}$	(0.5877,0.2305,0.0943)	(0.6076,0.0899,0.2154)	(0.6005,0.1693,0.1444)	(0.6005,0.1070,0.1591)
${ℷ}_{3}$	(0.5333,0.2054,0.1280)	(0.6645,0.0545,0.1505)	(0.5989,0.1839,0.0786)	(0.5989,0.1028,0.1652)
${ℷ}_{4}$	(0.5609,0.0885,0.1423)	(0.5680,0.1805,0.0498)	(0.5812,0.1263,0.0731)	(0.5812,0.1968,0.1123)
${ℷ}_{5}$	(0.5561,0.1296,0.1448)	(0.5687,0.1768,0.0882)	(0.5800,0.1763,0.0616)	(0.5800,0.1775,0.0644)

**Step 5:** Compute supports by using the consequences of Table 4, Table 5. For this purpose, we use Equations (16), (17), (18). The importance degree of weighted support Y and $Y^{\prime }$ produced for the PFAAPWA and PFAAPWG operators respectively.

$$Y=\left(\begin{array}{cc} \begin{array}{ccc} 0.3380 & 0.2968 & 0.2535\\ 0.3366 & 0.2962 & 0.2560\\ \begin{array}{c} 0.3393\\ 0.3358\\ 0.3354 \end{array} & \begin{array}{c} 0.2930\\ 0.2979\\ 0.2990 \end{array} & \begin{array}{c} 0.2568\\ 0.2566\\ 0.2553 \end{array} \end{array} & \begin{array}{c} 0.1117\\ 0.1113\\ \begin{array}{c} 0.1109\\ 0.1097\\ 0.1102 \end{array} \end{array} \end{array}\right)$$

And

$$Y^{\prime }=\left(\begin{array}{cc} \begin{array}{ccc} 0.3360 & 0.2977 & 0.2552\\ 0.3358 & 0.2962 & 0.2567\\ \begin{array}{c} 0.3372\\ 0.3360\\ 0.3353 \end{array} & \begin{array}{c} 0.2956\\ 0.2973\\ 0.2983 \end{array} & \begin{array}{c} 0.2560\\ 0.2560\\ 0.2554 \end{array} \end{array} & \begin{array}{c} 0.1111\\ 0.1113\\ \begin{array}{c} 0.1112\\ 0.1108\\ 0.1110 \end{array} \end{array} \end{array}\right)$$

**Step 6:** Assessment of PF information, which is listed in Table 4, Table 5, by using the above-given expression in Equation (19) and Equation (20). Computed results are stated in Table 6.

**Table 6:** Derived results from PFAAPWA and PFAAPWG operators.

PFAAPWA	PFAAPWG
(0.0821,0.7935,0.7134)	(0.8027,0.0712,0.0433)
(0.0686,0.7913,0.7665)	(0.8000,0.0736,0.0689)
(0.0729,0.7730,0.7538)	(0.7975,0.0664,0.0571)
(0.0948,0.7462,0.7161)	(0.7836,0.0627,0.0422)
(0.0652,0.7918,0.7184)	(0.7825,0.0735,0.0440)

**Step 7:** Compute the results of score values by using Equation (1). Because there is no need to find the accuracy values of Equation (2). Table 7 contains the results of score values by the PFAAPWA and PFAAPWG operators.

**Table 7:** Obtained score values from the PFAAPWA and PFAAPWG operators.

	$S\left({ℷ}_{1}\right)$	$S\left({ℷ}_{2}\right)$	$S\left({ℷ}_{3}\right)$	$S\left({ℷ}_{4}\right)$	$S\left({ℷ}_{5}\right)$	Ranking and ordering
PFAAPWA	(0.1918)	(0.1703)	(0.1820)	(0.2109)	(0.1850)	${ℷ}_{4}>{ℷ}_{1}>{ℷ}_{5}>{ℷ}_{3}>{ℷ}_{2}$
PFAAPWG	(0.8961)	(0.8858)	(0.8913)	(0.8929)	(0.8883)	${ℷ}_{1}>{ℷ}_{4}>{ℷ}_{3}>{ℷ}_{5}>{ℷ}_{2}$

**Fig. 1:**
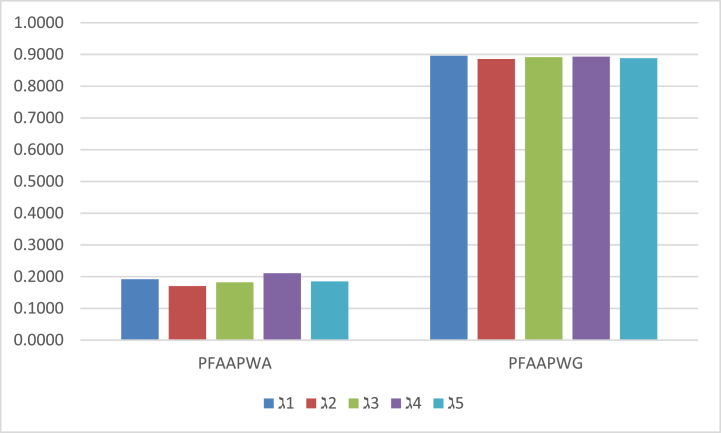
Explores the results of score values by the PFAAPWA and PFAAPWG operators.

Finally, we have to analyze more appropriate alternatives by ranking and ordering the score values. By observing the ranking and ordering of score values, we investigate ${ℷ}_{4}$ and ${ℷ}_{1}$ are more prominent alternatives by the PFAAPWA and PFAAPWG operators, respectively. We also study the behaviour of computed score values in a graphical representation of Fig. 1.

### 6.4 Sensitivity analysis

**Fig. 2:**
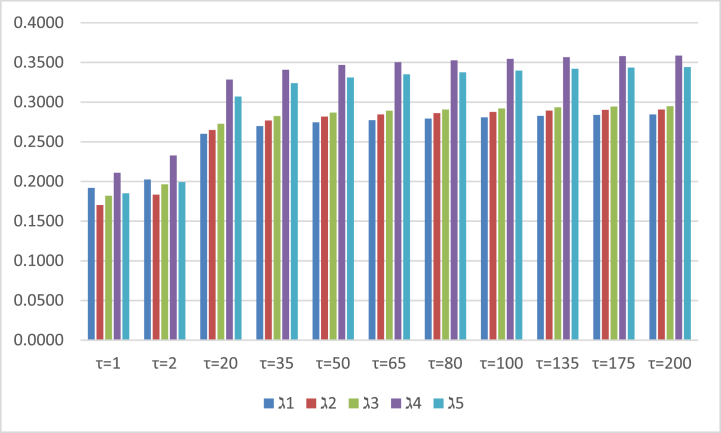
Covers the results of the score values, which are investigated by setting different parametric values in the PFAAPWA operator.

**Fig. 3:**
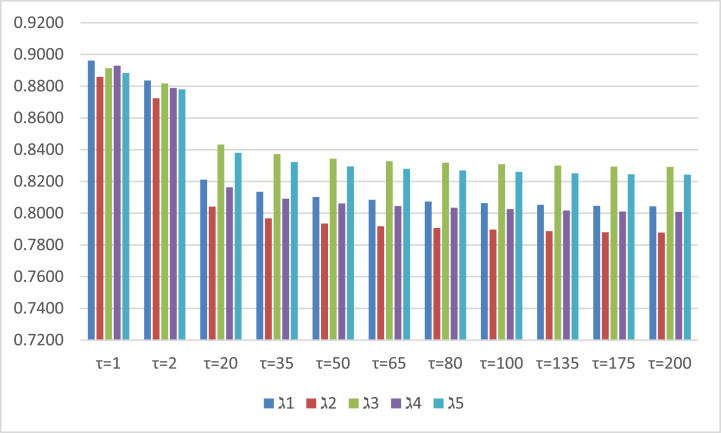
Covers the results of the score values, which are investigated by setting different parametric values in the PFAAPWA operator.

To examine the superiority and supremacy of our derived approaches. By setting different parametric values of Aczel Alsina aggregation tools in proposed methodologies after the aggregation process by the PFAAPWA and PFAAPWG operators. We produced results of score values from the PFAAPWA and PFAAPWG operators by changing different parametric values of τ. We adopted the results of score values by the PFAAPWA and PFAAPWG operators and stated them in Table 8 and Table 9, respectively. After the estimation of the score values which are shown in Table 8, the outcomes of the PFAAPWA operator are gradually increased if the parametric value of τ maximizes and the ranking of score values remains the same at τ>2. We also observed the results of score values obtained from the PFAAPWG operator, shown in Table 9. From Tables 9 and if the parametric values of τ are maximized, then the results of score values begin to decrease, and the ranking of score values remains unchanged at τ>2. We also exposed the results of score values in graphical representations of Fig. 2, Fig. 3, which are listed in Table 8, Table 9 respectively.

**Table 8:** Arranges the aggregated outcome of the PFAAPWA operator.

	$S\left({ℷ}_{1}\right)$	$S\left({ℷ}_{2}\right)$	$S\left({ℷ}_{3}\right)$	$S\left({ℷ}_{4}\right)$	$S\left({ℷ}_{5}\right)$	Ranking and ordering
τ=1	(0.1918)	(0.1703)	(0.1820)	(0.2109)	(0.1850)	${ℷ}_{4}>{ℷ}_{1}>{ℷ}_{5}>{ℷ}_{3}>{ℷ}_{2}$
τ=2	(0.2023)	(0.1833)	(0.1963)	(0.2328)	(0.1990)	${ℷ}_{4}>{ℷ}_{1}>{ℷ}_{5}>{ℷ}_{3}>{ℷ}_{2}$
τ=20	(0.2599)	(0.2647)	(0.2726)	(0.3283)	(0.3068)	${ℷ}_{4}>{ℷ}_{5}>{ℷ}_{3}>{ℷ}_{2}>{ℷ}_{1}$
τ=35	(0.2698)	(0.2767)	(0.2824)	(0.3407)	(0.3237)	${ℷ}_{4}>{ℷ}_{5}>{ℷ}_{3}>{ℷ}_{2}>{ℷ}_{1}$
τ=50	(0.2744)	(0.2817)	(0.2866)	(0.3467)	(0.3310)	${ℷ}_{4}>{ℷ}_{5}>{ℷ}_{3}>{ℷ}_{2}>{ℷ}_{1}$
τ=65	(0.2772)	(0.2844)	(0.2890)	(0.3502)	(0.3349)	${ℷ}_{4}>{ℷ}_{5}>{ℷ}_{3}>{ℷ}_{2}>{ℷ}_{1}$
τ=80	(0.2790)	(0.2861)	(0.2906)	(0.3525)	(0.3375)	${ℷ}_{4}>{ℷ}_{5}>{ℷ}_{3}>{ℷ}_{2}>{ℷ}_{1}$
τ=100	(0.2807)	(0.2876)	(0.2920)	(0.3545)	(0.3397)	${ℷ}_{4}>{ℷ}_{5}>{ℷ}_{3}>{ℷ}_{2}>{ℷ}_{1}$
τ=135	(0.2826)	(0.2891)	(0.2934)	(0.3566)	(0.3420)	${ℷ}_{4}>{ℷ}_{5}>{ℷ}_{3}>{ℷ}_{2}>{ℷ}_{1}$
τ=175	(0.2838)	(0.2902)	(0.2944)	(0.3580)	(0.3435)	${ℷ}_{4}>{ℷ}_{5}>{ℷ}_{3}>{ℷ}_{2}>{ℷ}_{1}$
τ=200	(0.2843)	(0.2906)	(0.2948)	(0.3586)	(0.3441)	${ℷ}_{4}>{ℷ}_{5}>{ℷ}_{3}>{ℷ}_{2}>{ℷ}_{1}$

**Table 9:** Arranges the aggregated outcome of the PFAAPWG operator.

	$S\left({ℷ}_{1}\right)$	$S\left({ℷ}_{2}\right)$	$S\left({ℷ}_{3}\right)$	$S\left({ℷ}_{4}\right)$	$S\left({ℷ}_{5}\right)$	Ranking and ordering
τ=1	(0.8960)	(0.8858)	(0.8913)	(0.8929)	(0.8883)	${ℷ}_{1}>{ℷ}_{4}>{ℷ}_{3}>{ℷ}_{5}>{ℷ}_{2}$
τ=2	(0.8835)	(0.8724)	(0.8817)	(0.8789)	(0.8780)	${ℷ}_{1}>{ℷ}_{3}>{ℷ}_{4}>{ℷ}_{5}>{ℷ}_{2}$
τ=20	(0.8211)	(0.8841)	(0.8432)	(0.8163)	(0.8380)	${ℷ}_{2}>{ℷ}_{3}>{ℷ}_{5}>{ℷ}_{1}>{ℷ}_{4}$
τ=35	(0.8134)	(0.7967)	(0.8372)	(0.8091)	(0.8321)	${ℷ}_{3}>{ℷ}_{5}>{ℷ}_{4}>{ℷ}_{1}>{ℷ}_{2}$
τ=50	(0.8102)	(0.7935)	(0.8344)	(0.8061)	(0.8294)	${ℷ}_{3}>{ℷ}_{5}>{ℷ}_{1}>{ℷ}_{4}>{ℷ}_{2}$
τ=65	(0.8084)	(0.7917)	(0.8328)	(0.8045)	(0.8279)	${ℷ}_{3}>{ℷ}_{5}>{ℷ}_{1}>{ℷ}_{4}>{ℷ}_{2}$
τ=80	(0.8073)	(0.7906)	(0.8318)	(0.8034)	(0.8269)	${ℷ}_{3}>{ℷ}_{5}>{ℷ}_{1}>{ℷ}_{4}>{ℷ}_{2}$
τ=100	(0.8063)	(0.7897)	(0.8309)	0.8025	(0.8260)	${ℷ}_{3}>{ℷ}_{5}>{ℷ}_{1}>{ℷ}_{4}>{ℷ}_{2}$
τ=135	(0.8052)	(0.7886)	(0.8300)	(0.8016)	(0.8251)	${ℷ}_{3}>{ℷ}_{5}>{ℷ}_{1}>{ℷ}_{4}>{ℷ}_{2}$
τ=175	(0.8046)	(0.7880)	(0.8293)	(0.8010)	(0.8245)	${ℷ}_{3}>{ℷ}_{5}>{ℷ}_{1}>{ℷ}_{4}>{ℷ}_{2}$
τ=200	(0.8043)	(0.7877)	(0.8291)	(0.8007)	(0.8243)	${ℷ}_{3}>{ℷ}_{5}>{ℷ}_{1}>{ℷ}_{4}>{ℷ}_{2}$

## 7 Comparative analysis

**Fig. 4:**
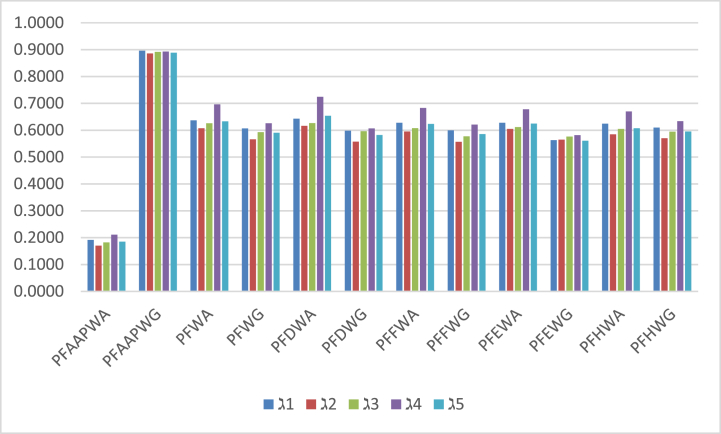
Explores the findings of existing approaches.

To verify the supremacy and validity of our proposed methods, we check the performance of currently discussed approaches with existing methodologies under consideration in the practical case study which is evaluated in the previous section. We applied different existing AOs and obtained results after the assessment of the practical case study. AOs of PF information based on some appropriate degrees of weights Wei [58], Jana et al. [63] produced some AOs of PFSs based on Dombi aggregation tools, Seikh and Mandal [64], some approaches of Frank aggregation tools under PF information are presented by the Khan et al. [65], AOs of Hamacher aggregation tools based on PF information was developed by the Wei [55] and AOs of complex PFSs proposed by the Hussain et al. [56]. All computed outcomes are listed in Table 10. After examining the results of existing aggregation techniques, all existing approaches cannot abolish the influence of unfair objects in the decision-making process. We concluded that our current derived approaches are more powerful and effective. The PFAAPWA and PFAAPWG operators can define correlation among different arguments. In Fig. 4, we illustrated the results of score values produced by the existing mythologies and revealed the geometrical behavior of current results and existing approaches.

**Table 10:** Covers the results of the comparative study.

AOs	Score values	Ranking and ordering
PFAAPWA	$\begin{array}{c} S\left({ℷ}_{1}\right)=0.1918,S\left({ℷ}_{2}\right)=0.1703\text{,}\\ S\left({ℷ}_{3}\right)=0.1820,S\left({ℷ}_{4}\right)=0.2109\\ ,S\left({ℷ}_{5}\right)=0.1850 \end{array}$	${ℷ}_{4}>{ℷ}_{1}>{ℷ}_{5}>{ℷ}_{3}>{ℷ}_{2}$
PFAAPWG	$S\left({ℷ}_{1}\right)=0.8961,S\left({ℷ}_{2}\right)=0.8858\text{,}$ $S\left({ℷ}_{3}\right)=0.8913,S\left({ℷ}_{4}\right)=0.8929\text{,}$ $S\left({ℷ}_{5}\right)=0.8883$	${ℷ}_{1}>{ℷ}_{4}>{ℷ}_{3}>{ℷ}_{5}>{ℷ}_{2}$
PFWA [58]	$S\left({ℷ}_{1}\right)=0.6367,S\left({ℷ}_{2}\right)=0.6072\text{,}$ $S\left({ℷ}_{3}\right)=0.6259,S\left({ℷ}_{4}\right)=0.6958\text{,}$ $S\left({ℷ}_{5}\right)=0.6328$	${ℷ}_{4}>{ℷ}_{1}>{ℷ}_{5}>{ℷ}_{3}>{ℷ}_{2}$
PFWG [58]	$S\left({ℷ}_{1}\right)=0.6064,S\left({ℷ}_{2}\right)=0.5658\text{,}$ $S\left({ℷ}_{3}\right)=0.5927,S\left({ℷ}_{4}\right)=0.6259\text{,}$ $S\left({ℷ}_{5}\right)=0.5904$	${ℷ}_{4}>{ℷ}_{1}>{ℷ}_{3}>{ℷ}_{5}>{ℷ}_{2}$
PFDWA [63]	$S\left({ℷ}_{1}\right)=0.6430,S\left({ℷ}_{2}\right)=0.6160\text{,}$ $S\left({ℷ}_{3}\right)=0.6261,S\left({ℷ}_{4}\right)=0.7243\text{,}$ $S\left({ℷ}_{5}\right)=0.6536$	${ℷ}_{4}>{ℷ}_{5}>{ℷ}_{1}>{ℷ}_{3}>{ℷ}_{2}$
PFDWG [63]	$S\left({ℷ}_{1}\right)=0.5982,S\left({ℷ}_{2}\right)=0.5573\text{,}$ $S\left({ℷ}_{3}\right)=0.5958,S\left({ℷ}_{4}\right)=0.6066\text{,}$ $S\left({ℷ}_{5}\right)=0.5818$	${ℷ}_{4}>{ℷ}_{1}>{ℷ}_{3}>{ℷ}_{5}>{ℷ}_{2}$
PFFWA [64]	$S\left({ℷ}_{1}\right)=0.6272,S\left({ℷ}_{2}\right)=0.5948\text{,}$ $S\left({ℷ}_{3}\right)=0.6073,S\left({ℷ}_{4}\right)=0.6825\text{,}$ $S\left({ℷ}_{5}\right)=0.6232$	${ℷ}_{4}>{ℷ}_{1}>{ℷ}_{5}>{ℷ}_{3}>{ℷ}_{2}$
PFFWG [64]	$S\left({ℷ}_{1}\right)=0.5994,S\left({ℷ}_{2}\right)=0.5565\text{,}$ $S\left({ℷ}_{3}\right)=0.5772,S\left({ℷ}_{4}\right)=0.6207\text{,}$ $S\left({ℷ}_{5}\right)=0.5856$	${ℷ}_{4}>{ℷ}_{1}>{ℷ}_{5}>{ℷ}_{3}>{ℷ}_{2}$
PFEWA [65]	$S\left({ℷ}_{1}\right)=0.6277,S\left({ℷ}_{2}\right)=0.6049\text{,}$ $S\left({ℷ}_{3}\right)=0.6113,S\left({ℷ}_{4}\right)=0.6778\text{,}$ $S\left({ℷ}_{5}\right)=0.6246$	${ℷ}_{4}>{ℷ}_{1}>{ℷ}_{5}>{ℷ}_{3}>{ℷ}_{2}$
PFEWG [65]	$S\left({ℷ}_{1}\right)=0.5629,S\left({ℷ}_{2}\right)=0.5647\text{,}$ $S\left({ℷ}_{3}\right)=0.5764,S\left({ℷ}_{4}\right)=0.5816\text{,}$ $S\left({ℷ}_{5}\right)=0.5604$	${ℷ}_{4}>{ℷ}_{3}>{ℷ}_{2}>{ℷ}_{1}>{ℷ}_{5}$
PFHWA [55]	$S\left({ℷ}_{1}\right)=0.6238,S\left({ℷ}_{2}\right)=0.5845\text{,}$ $S\left({ℷ}_{3}\right)=0.6047,S\left({ℷ}_{4}\right)=0.6695\text{,}$ $S\left({ℷ}_{5}\right)=0.6072$	${ℷ}_{4}>{ℷ}_{1}>{ℷ}_{5}>{ℷ}_{3}>{ℷ}_{2}$
PFHWG [55]	$S\left({ℷ}_{1}\right)=0.6096,S\left({ℷ}_{2}\right)=0.5699\text{,}$ $S\left({ℷ}_{3}\right)=0.5946,S\left({ℷ}_{4}\right)=0.6335\text{,}$ $S\left({ℷ}_{5}\right)=0.5948$	${ℷ}_{4}>{ℷ}_{1}>{ℷ}_{5}>{ℷ}_{3}>{ℷ}_{2}$
Hussain et al. [56]	Not applicable	
Mahmood and Ahmmad [57]	Not applicable	
Mahmood et al. [66]	Not applicable

We observed some existing approaches cannot handle the given information seen in Refs. [[56], [57], [66]] due to insufficient information. After deep analysis, we concluded our proposed research work is more applicable and effective due to the parametric values of Aczel Alsina aggregation tools. By using these parametric values, decision-makers can achieve results according to their preferences. This presented research work provides smooth approximation by using concepts of unknown degrees of weight and reduces the loss of information during decision-making.

## 8 Conclusion

The MAGDM process is used to evaluate a realistic optimal option using different aggregation methodologies. AOs are essential to aggregate given information in a specific single term. For this purpose, PFSs are an efficient aggregation model, which is the extended form of FSs and IFSs. In this article, we exposed the theory of power operators to mitigate the impact of unpredictable and redundant information on human opinions. Some robust operations of Aczel Alsina aggregation tools were demonstrated in the light of PF information. Aczel Alsina tools have recently become more attractive aggregation models, and numerous scholars have worked on different fuzzy domains. The key points of the proposed research work are listed as follows:

a) We exposed notions of PFSs and their related fundamental operations.
b) Some reliable operations of Aczel Alsina aggregation tools were developed in light of PF information.
c) By utilizing the concepts of power operators, we derived a class of new approaches under considering PF environments, including PFAAPWA, PFAAPOWA, PFAAPWG, and PFAAPWG operators.
d) An algorithm for the MAGDM problem is also illustrated based on PF information to find a suitable optimal option.
e) Additionally, a numerical example demonstrated selecting a reliable air conditioner under certain characteristics.
f) To illustrate the superiority of our proposed approaches, a comprehensive comparative study is also presented to compare the findings of existing approaches with currently discussed AOs.

Sometimes, our invented methodologies are unable to deal with redundant or vague information about any object. We will extend our proposed research work to handle this situation in different fuzzy frameworks like special fuzzy sets and t-spherical fuzzy sets. We also try to resolve some complicated challenges such as pattern recognition, structure development, green-supplied systems, similarity measures, medical diagnosis, and improvement in the construction sector. In the coming future, we will extend our developed research work in different fuzzy domains such as bipolar soft set [67], bipolar complex fuzzy set [68], complex spherical fuzzy set [69], 3,4-quasirung FSs [70], quasirung orthopair fuzzy sets [71], Z-fuzzy sets [72], Decomposed Fuzzy sets [73] and m-polar fuzzy sets [74], moreover, the proposed model can be integrated with different decision making models [[75], [76], [77]].

## Contributions

All authors contributed equally to preparing this manuscript.

## Data availability statement

Data will be made available on request.

## Ethical approval

This article does not contain any studies with human participants or animals performed by the author.

## Funding

This article was partially funded by the 10.13039/501100001602Science Foundation Ireland (10.13039/501100001602SFI) through the Digital Voting Hub for Sustainable Urban Transport System, VOTE-TRA project (22/NCF/DR/11309).

## CRediT authorship contribution statement

**Abrar Hussain:** Writing – original draft, Methodology, Formal analysis, Conceptualization. **Yu Liu:** Writing – review & editing, Visualization, Conceptualization. **Kifayat Ullah:** Writing – original draft, Formal analysis, Data curation, Conceptualization. **Muhammad Rashid:** Writing – original draft, Resources, Methodology, Conceptualization. **Tapan Senapati:** Writing – review & editing, Visualization, Supervision, Conceptualization. **Sarbast Moslem:** Writing – review & editing, Visualization, Validation, Supervision, Conceptualization.

## Declaration of competing interest

The authors declare that they have no known competing financial interests or personal relationships that could have appeared to influence the work reported in this paper.
